# Emerging Porous Materials and Their Composites for NH_3_ Gas Removal

**DOI:** 10.1002/advs.202002142

**Published:** 2020-11-01

**Authors:** Dong Won Kang, Susan Eungyung Ju, Dae Won Kim, Minjung Kang, Hyojin Kim, Chang Seop Hong

**Affiliations:** ^1^ Department of Chemistry Korea University Seoul 02841 Republic of Korea

**Keywords:** composite materials, covalent organic frameworks, metal–organic frameworks, NH_3_ adsorbents, porous organic polymers

## Abstract

NH_3_, essential for producing artificial fertilizers and several military and commercial products, is being produced at a large scale to satisfy increasing demands. The inevitable leakage of NH_3_ during its utilization, even in trace concentrations, poses significant environmental and health risks because of its highly toxic and reactive nature. Although numerous techniques have been developed for the removal of atmospheric NH_3_, conventional NH_3_ abatement systems possess the disadvantages of high maintenance cost, low selectivity, and emission of secondary wastes. In this context, highly tunable porous materials such as metal–organic frameworks, covalent organic frameworks, hydrogen organic frameworks, porous organic polymers, and their composite materials have emerged as next‐generation NH_3_ adsorbents. Herein, recent progress in the development of porous NH_3_ adsorbents is summarized; furthermore, factors affecting NH_3_ capture are analyzed to provide a reasonable strategy for the design and synthesis of promising materials for NH_3_ abatement.

## Graphical abstract

This review affords a comprehensive overview of porous materials and their composites to capture NH_3_ gas.

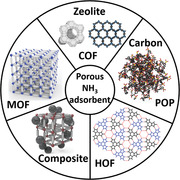

## 1 Introduction

Undoubtedly, NH_3_ has become part of our daily lives. Ever since Nobel Laureates Fritz Haber and Carl Bosch demonstrated nitrogen fixation to produce NH_3_ more than a century ago, artificial fertilizers have saved the ever‐increasing human population from starvation. Agriculture accounts for 80% of the total consumption of NH_3_; however, NH_3_ is also used to develop military and commercial products, including but not limited to explosives, refrigerants, pharmaceuticals, plastics, synthetic fibers, and cleaning agents.^[^ ^1^, ^2^, ^3^, ^4^ ^]^

The high hydrogen density of NH_3_ makes it a promising hydrogen carrier for various fuel cells, such as alkaline fuel cells, phosphoric acid fuel cells, and polymer electrolyte membrane fuel cells.^[^ ^5^ ^]^ The carbon‐free nature of NH_3_ also enticed researchers to investigate its usage as an alternative energy source in solid oxide fuel cells.^[^ ^6^ ^]^ Amid elevated demands, the annual global production of NH_3_ reached ≈150 million metric tons, and this amount is expected to grow by 4% in the next 4 years.^[^ ^1^ ^]^

The unavoidable leakage of NH_3_ during its utilization has huge adverse impact on the environment and human health. This colorless and corrosive gas, with a pungent odor and high vapor pressure (1003 kPa at 25 °C), is detrimental to health even in trace concentrations. Direct exposure to NH_3_ causes irritation, mainly to the eyes, skin, and respiratory system.^[^ ^7^ ^]^ Thus, the Occupational Safety and Health Administration (OSHA) recently revised the limit for NH_3_ from 50 ppm as an 8 h total weight average (TWA) to 35 ppm as a short‐term exposure limit (STEL).^[^ ^4^ ^]^ Moreover, the emitted NH_3_ reacts with the nitrogen and sulfur oxides (NO*_x_* and SO*_x_*) present in air to form fine particulate matter with a diameter less than 2.5 µm (PM2.5); such particulate matter is known to trigger premature death.^[^ ^8^ ^]^ Furthermore, an increased level of NH_3_ in the atmosphere contributes to the acidification of coastal water and disrupts aquatic life.^[^ ^9^ ^]^ Stringent control of NH_3_ is also crucial in laboratory and industrial settings. While NH_3_ is an unavoidable contaminant in proton exchange membrane fuel cells even when it is not an initial source, the performance of such cells is strikingly impaired in the presence of NH_3_.^[^ ^10^ ^]^ During photolithography, airborne NH_3_ should be monitored and maintained below parts per billion by volume (ppbv) level to ensure the integrity of the experiments.^[^ ^11^ ^]^

Thus, owing to the critical risks associated with this widely used gas, effective means to capture and store NH_3_ have garnered substantial attention. To date, numerous techniques to remove atmospheric NH_3_ has been developed.^[^ ^12^ ^]^ Traditionally, NH_3_ from industrial gas stream has mainly been retrieved with dilute sulfuric acid in the form of ammonium sulfate.^[^ ^13^, ^14^ ^]^ However, as the market demand of ammonium sulfate diminished, other processes such as Phosam process or Chevron wastewater treatment have been employed to collect NH_3_.^[^ ^15^, ^16^ ^]^ Also, direct combustion or usage of heterogenous catalysts to induce the catalytic oxidation of NH_3_ into N_2_, NO*_x_*, and H_2_O are commonly used to remove NH_3_ gas.^[^ ^17^, ^18^ ^]^ To abate NH_3_ gas resulting from agricultural activities, scrubbers, biological substances, and membranes are currently utilized.^[^ ^19^, ^20^ ^]^ For instance, the scrubbing process efficiently dissolves the gas by promoting contact with water droplets. Biological nitrification by NH_3_‐specific microorganisms has been investigated using bioscrubbers, biofilters, and biotrickling filters.^[^ ^21^ ^]^ In continuous flux conditions, selectively permeable membranes are promising tools to cost‐effectively separate NH_3_ from air.^[^ ^22^ ^]^ However, such conventional NH_3_ abatement systems have limitations of high maintenance cost, low selectivity, or generation of secondary wastes such as biomass or NO*_x_*.

Solid adsorbents such as polymer resins, silica gels, alumina, zeolites, and carbonaceous materials including activated carbons, charcoals and activated carbon fibers, which operate under mild conditions, have been the subject of extensive studies.^[^ ^23^, ^24^, ^25^ ^]^ When the NH_3_ adsorption capacities of the adsorbents were compared, zeolites showed higher NH_3_ uptake amounts than polymer resins, silica gels, and alumina under the same conditions.^[^ ^26^ ^]^ Since the pure forms of the adsorbents exhibited low selectivity of NH_3_, methods to selectively bind NH_3_ have been studied. For instance, impregnation of porous alumina with alkaline‐earth metal chlorides increased dynamic capacity, presumably due to the formation of an NH_3_ complex resulting from salt–ammonia interaction, although the NH_3_ removal capability still fell behind that of zeolites.^[^ ^27^ ^]^ Among the various candidates, activated carbons are prevalently incorporated in commercial filters as inexpensive sorbents.^[^ ^26^, ^28^, ^29^, ^30^, ^31^, ^32^, ^33^ ^]^ As activated carbons bind NH_3_ via predominantly weak attractive forces, further oxidation or impregnation of the carbon materials with metal chlorides or metal oxides has been examined to provide additional forces.^[^ ^34^, ^35^ ^]^ Although surface modification aids adsorption to a certain extent, sophisticated designs are limited by inherently irregular pore sizes and the functionality of the amorphous network. Similarly, acid functionalization on zeolites did not significantly increase the adsorption capacity and the reproduction of the target materials was difficult.^[^ ^36^ ^]^ In addition, the structures of zeolites are prone to collapse when the pore size increases.^[^ ^37^ ^]^

**Figure 1:**
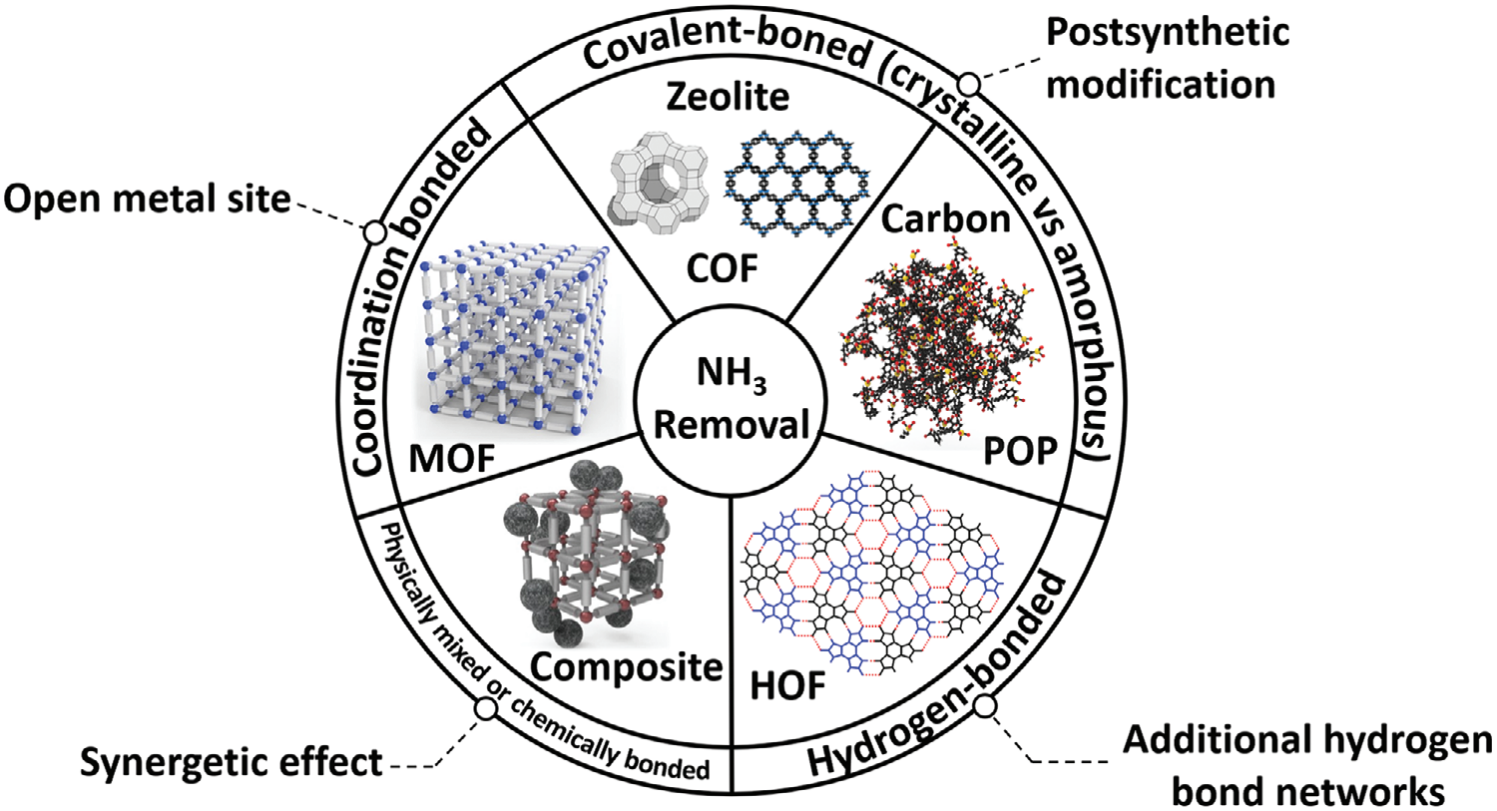
Classification of porous NH_3_ adsorbents, and their desirable characteristics for NH_3_ capture.

In this context, highly tunable porous materials such as metal–organic frameworks (MOFs), covalent organic frameworks (COFs), hydrogen‐bonded organic frameworks (HOFs), and porous organic polymers (POPs) have emerged as next‐generation NH_3_ adsorbents (**Figure** **1**).^[^ ^38^, ^39^, ^40^, ^41^, ^42^ ^]^ Owing to the high porosity and tunable functionality, which are unique to the porous materials, each structure can be tailored to possess top‐notch NH_3_ adsorption capacity or improved durability.^[^ ^12^, ^43^ ^]^ MOFs, which consist of metal clusters bridged by organic spacers, are the most actively investigated inorganic–organic hybrid material. More recently, porous frameworks and polymers constructed with covalent bonds (COFs and POPs) and hydrogen bonds (HOFs) between the organic elements were reported to selectively bind NH_3_. The large surface areas, diverse topologies, and versatile pore structures of the porous materials contribute to their diverse applications, such as gas storage and separation, catalysis, magnetism, proton conduction, and chemical sensing.^[^ ^44^, ^45^, ^46^, ^47^ ^]^ In fact, products that exhibit a large adsorption capacity and reversibility could potentially be a solution for the safe storage of NH_3_. The specific properties needed for an intended application could be readily obtained by altering the constituents or postsynthetic procedures. For instance, as NH_3_ acts both as a Lewis base and a Brønsted base, acidic sites on a porous platform could enhance its capture at low concentrations. Such acidic sites could be achieved by removing the solvent molecules on the nodes of MOFs to generate open metal sites with high Lewis acidity;^[^ ^48^ ^]^ incorporating acidic functional groups such as carboxylic acid, phosphoric acid, and sulfonic acid groups to POPs;^[^ ^49^ ^]^ or introducing metal ions to COFs.^[^ ^50^ ^]^ In the postsynthetic approach, a variety of acidic groups, such as carboxylic acid or sulfonic acid groups, could be functionalized on the pore surface to promote the chemisorption of the basic gas. Additionally, composite materials, obtained by mixing porous materials and other materials such as polymer or activated carbons, have recently become one of the excellent alternatives for developing effective materials.^[^ ^51^, ^52^, ^53^ ^]^ This method is significantly attractive in that it can easily compensate for the shortcomings of each material, enabling better performance.

To facilitate the development of efficient NH_3_ adsorbents, several reviews describing the advancement of NH_3_ abatement systems have been published. However, they primarily discuss MOFs, and newly studied porous frameworks and polymers have not been discussed.^[^ ^12^, ^43^, ^44^, ^54^, ^55^, ^56^, ^57^, ^58^ ^]^ Herein, we present the recent progress in emerging porous materials for NH_3_ capture mainly focusing on metal–organic frameworks, covalent organic frameworks, and porous organic polymers (**Table** **1**). The goal of this review is to provide a better understanding of the variables that influence NH_3_ adsorption and to establish guiding principles for the design of materials that possess desired traits.

**Table 1:** Performances of reported NH_3_ adsorbents (GO: graphite oxide; PVDF: polyvinylidene fluoride)

Metal–organic frameworks						
Compounds	Measurement type	Adsorbed amounts [mmol g^−1^]	Analysis condition	Prominent features	Activation/regeneration condition	Refs.
3D‐[Zn_2_(L1)_2_(bipy)]	Isotherm	14.3	0 °C, 1000 mbar	Free urea groups	40 °C, 4 h, vacuum/–	^[^ ^83^ ^]^
3D‐[Zn_2_(L1)_2_(bpe)]	Isotherm	17.8	0 °C, 1000 mbar	Free urea groups	40 °C, 4 h, vacuum/–	
NU‐300	Isotherm	8.28	25 °C, 1000 mbar	Free carboxylic acid groups	120 °C, overnight, vacuum/RT, vacuum	^[^ ^88^ ^]^
		4	25 °C, 0.1 mbar			
		1.5	25 °C, 0.01 mbar			
Ga‐PMOF	Isotherm	10.50	25 °C, 1000 mbar	Brønsted acidic bridging hydroxyl group	140 °C, 12 h/without thermal activation	^[^ ^89^ ^]^
In‐PMOF	Isotherm	9.41	25 °C, 1000 mbar	Brønsted acidic bridging hydroxyl group	140 °C, 12 h/without thermal activation	
Al‐PMOF	Isotherm	7.67	25 °C, 1000 mbar	Brønsted acidic bridging hydroxyl group	140 °C, 12 h/without thermal activation	
	Breakthrough	0.29	25 °C, 0.5 mbar, 0% RH		170 °C, vacuum/–	^[^ ^69^ ^]^
		0.52	25 °C, 0.5 mbar, 80% RH			
Al‐PMOF‐HCl	Breakthrough	2.70	25 °C, 0.5 mbar, 0% RH	HCl loaded in the pores	170 °C, vacuum/–	
		4.63	25 °C, 0.5 mbar, 80% RH			
Al‐PMOF‐FA	Breakthrough	2.23	25 °C, 0.5 mbar, 0% RH	Formic acid loaded in the pores	170 °C, vacuum/–	
		3.22	25 °C, 0.5 mbar, 80% RH			
NU‐1401	Isotherm	8.41	25 °C, 1000 mbar	Electron‐deficient naphthalene diimide units, acidic Zr node	supercritical CO_2_ activation/–	^[^ ^90^ ^]^
	Breakthrough	5.7	20 °C, 2.97 mbar, 80% RH			
UiO‐66‐A	Isotherm	5.74	25 °C, 1000 mbar	Free —NH_2_ and —NH_3_^+^Cl^−^ groups	120 °C, 48 h, vacuum/–	^[^ ^65^ ^]^
UiO‐66‐B	Isotherm	6.81	25 °C, 1000 mbar	Postsynthetic hemiaminal functionalization	25 °C, 24 h, vacuum/–	
UiO‐66‐C	Isotherm	8.27	25 °C, 1000 mbar	Postsynthetic aziridine functionalization	85 °C, vacuum/–	
Mn_2_Cl_2_BTDD	Isotherm	15.47	25 °C, 1000 mbar	Open metal sites	100 °C, 24 h, vacuum/200 °C, vacuum	^[^ ^81^ ^]^
Co_2_Cl_2_BTDD	Isotherm	12.00	25 °C, 1000 mbar	Open metal sites	100 °C, 24 h, vacuum/200 °C, vacuum	
Ni_2_Cl_2_BTDD	Isotherm	12.02	25 °C, 1000 mbar	Open metal sites	100 °C, 24 h, vacuum/200 °C, vacuum	
Co_2_Cl_2_BBTA	Isotherm	17.95	25 °C, 1000 mbar	Open metal sites	150 °C, 24 h, vacuum/200 °C, vacuum	^[^ ^82^ ^]^
	Breakthrough	8.56	20 °C, 1 mbar, 0% RH		–/–	
		4.36	20 °C, 1 mbar, 80% RH			
Ni_2_Cl_2_BBTA	Isotherm	14.68	25 °C, 1000 mbar	Open metal sites	150 °C, 24 h, vacuum/–	
Cu_2_Cl_2_BBTA	Isotherm	19.79	25 °C, 1000 mbar	Open metal sites	150 °C, 24 h, vacuum/–	
	Breakthrough	7.52	20 °C, 1 mbar, 0% RH		–/–	
		5.73	20 °C, 1 mbar, 80% RH			
Co_2_Cl_2_BTDD	Breakthrough	4.78	20 °C, 1 mbar, 0% RH	Open metal sites	–/–	
		3.38	20 °C, 1 mbar, 80% RH			
Zn(INA)_2_	Isotherm	6	25 °C, 1000 mbar	Open metal sites	100 °C 1 h/120 °C, 2 h	^[^ ^70^ ^]^
Zn(NA)_2_	Isotherm	10.2	25 °C, 1000 mbar	Open metal sites, gate opening behavior	150 °C, overnight, vacuum/150 °C, 70 min, vacuum	^[^ ^71^ ^]^
Co(NA)_2_	Isotherm	17.5	25 °C, 1000 mbar	Open metal sites	200 °C, overnight, vacuum/150 °C, 70 min, vacuum	
Cu(NA)_2_	Isotherm	13.4	25 °C, 1000 mbar	Open metal sites	150 °C, overnight, vacuum/150 °C, 70 min, vacuum	
Cd(NA)_2_	Isotherm	6	25 °C, 1000 mbar	Open metal sites	150 °C, overnight, vacuum/150 °C, 70 min, vacuum	
DMOF	Breakthrough	0.27	20 °C, 1.44 mbar, 0% RH	–	–	^[^ ^67^ ^]^
		5.56	20 °C, 1.44 mbar, 80% RH			
CuBTB	Breakthrough	2.19	20 °C, 1.44 mbar, 0% RH	Open metal sites	–	
		5.95	20 °C, 1.44 mbar, 80% RH			
ZnBTTB	Breakthrough	4.59	20 °C, 1.44 mbar, 0% RH	Free carboxylic acid groups	250 °C, 2 h, vacuum/–	
		20.26	20 °C, 1.44 mbar, 80% RH			
DMOF‐A	Breakthrough	0.48	20 °C, 1.44 mbar, 0% RH	–	–	
		1.18	20 °C, 1.44 mbar, 80% RH			
DMOF‐TM2	Breakthrough	0.15	20 °C, 1.44 mbar, 0% RH	Free methyl groups	–	
		4.57	20 °C, 1.44 mbar, 80% RH			
ZIF‐8	Isotherm	1.2	25 °C, 1000 mbar	–	–/–	^[^ ^62^ ^]^
Al‐BTB	Isotherm	6.00	25 °C, 1000 mbar	–	–/–	
MIL‐53(Al)	Isotherm	4.28	25 °C, 1000 mbar	–	–/–	
MIL‐53	Isotherm	4.4	25 °C, 1000 mbar	–	330 °C, air/25 °C, 30 min, vacuum	^[^ ^68^ ^]^
NH_2_‐MIL‐53	Isotherm	8	25 °C, 1000 mbar	Free amino groups	30 °C, 24 h, vacuum/150 °C, 30 min, vacuum	
MIL‐100	Isotherm	8	25 °C, 1000 mbar	–	–/25 °C, 30 min, vacuum	
Al‐MIL‐101‐NH_2_	Breakthrough	1.70	25 °C, 1.2 mbar, 0% RH	Open metal sites	150 °C, 30 min, N_2_/–	
		2.28	25 °C, 1.2 mbar, 40% RH			
MIL‐101	Isotherm	10	25 °C, 1000 mbar	–	–/25 °C, 30 min, vacuum	
MFM‐300(Al)	Isotherm	13.9	20 °C, 1000 mbar	Brønsted acidic bridging hydroxyl groups	200 °C, 24 h, vacuum/vacuum	^[^ ^84^ ^]^
Fe‐BTC	Breakthrough	1.99	25 °C, 1.2 mbar, 0% RH	Meso‐porosity	150 °C, 30 min, N_2_/–	^[^ ^68^ ^]^
		2.34	25 °C, 1.2 mbar, 40% RH			
MOF‐199	Breakthrough	5.10	25 °C, 9.9 mbar	–	170 °C, 48 h, vacuum/–	^[^ ^59^ ^]^
IRMOF‐62	Breakthrough	1.35	25 °C, 9.9 mbar	–	150 °C, 27 h, vacuum/–	
IRMOF‐3	Breakthrough	6.16	25 °C, 9.9 mbar	–	120 °C, 23 h, vacuum/–	
Mg‐MOF‐74	Isotherm	16.2	25 °C, 1000 mbar	Open metal sites	–/–	^[^ ^62^ ^]^
	Breakthrough	7.60	20 °C, 1.44 mbar, 0% RH	Open metal sites	250 °C, 6 h, vacuum/–	^[^ ^61^ ^]^
		1.70	20 °C, 1.44 mbar, 80% RH			
Co‐MOF‐74	Breakthrough	6.70	20 °C, 1.44 mbar, 0% RH	Open metal sites	250 °C, 24 h, vacuum/–	
		4.30	20 °C, 1.44 mbar, 80% RH			
Ni‐MOF‐74	Breakthrough	2.30	20 °C, 1.44 mbar, 0% RH	Open metal sites	250 °C, 5 h, vacuum/–	
		1.90	20 °C, 1.44 mbar, 80% RH			
		3.22	25 °C, 1.2 mbar, 0% RH		150 °C, 30 min, N_2_/–	^[^ ^68^ ^]^
		3.40	25 °C, 1.2 mbar, 40% RH			
Cu‐MOF‐74	Breakthrough	3.4	2.88 mbar, 0% RH	Open metal sites	150 °C, 1 h, air/–	^[^ ^64^ ^]^
		7.6	2.88 mbar, 80% RH			
Zn‐MOF‐74	Breakthrough	3.70	20 °C, 1.44 mbar, 0% RH	Open metal sites	150 °C, 10 h + 265 °C, 10 h, vacuum/–	^[^ ^61^ ^]^
		2.80	20 °C, 1.44 mbar, 80% RH			
		2.75	25 °C, 1.2 mbar, 0% RH	Open metal sites	150 °C, 30 min, N_2_/–	^[^ ^68^ ^]^
		2.87	25 °C, 1.2 mbar, 40% RH			
		5.46	25 °C, 9.9 mbar	Open metal sites	150 °C, 10 h + 265 °C, 10 h, vacuum/–	^[^ ^59^ ^]^
UiO‐fumarate	Breakthrough	2.46	25 °C, 1.2 mbar, 0% RH	–	150 °C, 30 min, N_2_/–	^[^ ^68^ ^]^
		1.87	25 °C, 1.2 mbar, 40% RH			
UiO‐66‐COOH	Breakthrough	3.17	25 °C, 1.2 mbar, 0% RH	Free carboxylic acid groups	150 °C, 30 min, N_2_/–	
		3.17	25 °C, 1.2 mbar, 40% RH			
UiO‐66	Breakthrough	1.35	25 °C, 1.2 mbar, 0% RH	–	150 °C, 30 min, N_2_/–	
		1.52	25 °C, 1.2 mbar, 40% RH			
	Breakthrough	1.79	20 °C, 1.44 mbar, 0% RH	–	200 °C, overnight, vacuum/–	^[^ ^67^ ^]^
		2.75	20 °C, 1.44 mbar, 80% RH			
	Breakthrough	2.0	20 °C, 2.88 mbar	–	150 °C, 1 h, air/–	^[^ ^66^ ^]^
UiO‐66‐vac	Breakthrough	1.6	20 °C, 2.88 mbar	Missing linker	150 °C, 1 h, air/–	
UiO‐66‐ox	Breakthrough	2.5	20 °C, 2.88 mbar	Free carboxylic acid groups	150 °C, 1 h, air/–	
UiO‐66‐NH_2_	Breakthrough	1.40	25 °C, 1.2 mbar, 0% RH	Free amino groups	150 °C, 30 min, N_2_/–	^[^ ^68^ ^]^
		1.93	25 °C, 1.2 mbar, 40% RH			
		3.56	20 °C, 1.44 mbar, 0% RH		200 °C, overnight, vacuum/–	^[^ ^67^ ^]^
		3.01	20 °C, 1.44 mbar, 80% RH			
UiO‐66‐NO_2_	Breakthrough	1.98	20 °C, 1.44 mbar, 0% RH	Free nitro groups	170 °C, overnight, vacuum/–	
		1.60	20 °C, 1.44 mbar, 80% RH			
UiO‐66‐OH	Breakthrough	5.69	20 °C, 2.88 mbar, 0% RH	Free hydroxy groups	65 °C, overnight, vacuum/–	
		2.77	20 °C, 2.88 mbar, 80% RH			
UiO‐66‐(OH)_2_	Breakthrough	2.29	20 °C, 2.88 mbar, 0% RH	Free hydroxy groups	65 °C, overnight, vacuum/–	
		2.16	20 °C, 2.88 mbar, 80% RH			
UiO‐66‐SO_3_H	Breakthrough	2.24	20 °C, 2.88 mbar, 0% RH	Free sulfonic groups	65 °C, overnight, vacuum/–	
		1.45	20 °C, 2.88 mbar, 80% RH			
UiO‐66‐(COOH)_2_	Breakthrough	2.83	20 °C, 2.88 mbar, 0% RH	Free carboxylic acid groups	65 °C, overnight, vacuum/–	
		1.83	20 °C, 2.88 mbar, 80% RH			
Cu_3_(BTC)_2_	Isotherm	12.1	28 °C, 1200 mbar	Open metal sites	120 °C, 6 h, vacuum/–	^[^ ^121^ ^]^
		≈6.0	40 °C, 1000 mbar		120 °C, 15 h, vacuum/120 °C, vacuum	^[^ ^122^ ^]^
	Breakthrough	9.6	25 °C, 1.5 mbar		120 °C, 8 h, vacuum/–	^[^ ^111^ ^]^
		6.75	25 °C, 1 mbar, 0%		–/–	^[^ ^119^ ^]^
		10.09	25 °C, 1 mbar, humid			
		6.8	25 °C, 1 mbar		–/–	^[^ ^121^ ^]^
		7.39	25 °C, 1 mbar, 0% RH		120 °C, vacuum/–	^[^ ^124^ ^]^
		7.51	25 °C, 1 mbar, 70% RH			
		7.4	20 °C, 2.88 mbar		100 °C, 1 h, air/–	^[^ ^112^ ^]^
		6.6	20 °C, 1.44 mbar, 0% RH		100 °C, N_2_/100 °C, N_2_	^[^ ^63^ ^]^
		8.9	20 °C, 1.44 mbar, 80% RH			
		10.09	25 °C, 1 mbar, humid		130–135 °C, 6 h, vacuum/–	^[^ ^120^ ^]^
		5.34	25 °C, 1.2 mbar, 0% RH		150 °C, 30 min, N_2_/–	^[^ ^68^ ^]^
		6.57	25 °C, 1.2 mbar, 40% RH			
DUT‐6	Isotherm	12	25 °C, 1000 mbar	–	Supercritical CO_2_ activation/–	^[^ ^125^ ^]^
OH‐DUT‐6	Isotherm	16.4	25 °C, 1000 mbar	Free hydroxyl groups		
Fe‐MIL‐101‐SO_3_H	Isotherm	17.80	25 °C, 1000 mbar	Free sulfonic acid groups	125 °C, 24 h, vacuum/–	^[^ ^99^ ^]^
MOF‐5	Isotherm	12.2	25 °C, 1000 mbar	–	–/–	^[^ ^60^ ^]^
	Breakthrough	2.52	25 °C, 1 mbar, humid	–	130–135 °C, 6 h, vacuum/–	^[^ ^120^ ^]^
		0.34	25 °C, 1 mbar, 0% RH	– –	130–135 °C, 6 h, vacuum/–	^[^ ^117^ ^]^
		2.49	25 °C, 1 mbar, 70% RH			
		0.35	25 °C, 9.9 mbar	–	120 °C, 17 h, vacuum/–	^[^ ^59^ ^]^
MOF‐5‐E	Breakthrough	0.35	1 mbar	–	–/–	^[^ ^123^ ^]^
MOF‐177	Isotherm	12.2	25 °C, 1000 mbar	–	–/–	^[^ ^60^ ^]^
	Breakthrough	2.46	25 °C, 9.9 mbar	–	–/–	^[^ ^59^ ^]^
MIL‐ED	Breakthrough	3.27	25 °C, 1 mbar	MIL‐100(Fe)	120 °C, overnight, air	^[^ ^123^ ^]^
MIL‐EM	Breakthrough	2.3	25 °C, 1 mbar	MIL‐100(Fe)	120 °C, overnight, air	
MILd‐ED	Breakthrough	4.33	25 °C, 1 mbar	Dried MIL‐100(Fe)	120 °C, overnight, air,	
STAM‐17‐OEt	Breakthrough	2.54	25 °C, 0.45 mbar	Hemilabile bonds in the coordination environment	150 °C, overnight, vacuum	^[^ ^113^ ^]^
Other porous hybrid adsorbents						
ZSM‐5 (Si/Al: 23)	Breakthrough	2.23	25 °C, 1.2 mbar, 0% RH	Zeolite	250 °C, 30 min, N_2_/–	^[^ ^68^ ^]^
		1.46	25 °C, 1.2 mbar, 40% RH			
Y (Si/Al: 23)	Breakthrough	0.41	25 °C, 1.2 mbar, 0% RH	Zeolite	250 °C, 30 min, N_2_/–	
		0.41	25 °C, 1.2 mbar, 40% RH			
Y (Si/Al: 5.5)	Breakthrough	1.82	25 °C, 1.2 mbar, 0% RH	Zeolite	250 °C, 30 min, N_2_/–	
		0.70	25 °C, 1.2 mbar, 40% RH			
Beta	Breakthrough	1.40	25 °C, 1.2 mbar, 0% RH	Zeolite	250 °C, 30 min, N_2_/–	
		1.40	25 °C, 1.2 mbar, 40% RH			
Zeolite	Breakthrough	0.28	25 °C, 0.01 mbar	Zeolite	–/Water flushing	^[^ ^95^ ^]^
4A zeolite (Baylith TG242)	Isotherm	8.71	25 °C, 1000 mbar	Zeolite	300 °C, overnight/–	^[^ ^26^ ^]^
5A zeolite (Baylith KE154)	Isotherm	7.67	25 °C, 1000 mbar	Zeolite	300 °C, overnight/–	
5A zeolite (Sigma M‐5766)	Isotherm	7.43	25 °C, 1000 mbar	Zeolite	300 °C, overnight/–	
5A zeolite (Lancaster 5830)	Isotherm	7.81	25 °C, 1000 mbar	Zeolite	300 °C, overnight/–	
13X zeolite (Baylith WE894)	Isotherm	9.32	25 °C, 1000 mbar	Zeolite	300 °C, overnight/–	
13X zeolite (Lancaster 6149)	Isotherm	9.32	25 °C, 1000 mbar	Zeolite	300 °C, overnight/–	
13X zeolite (Sigma M‐3385)	Isotherm	9.03	25 °C, 1000 mbar	Zeolite	300 °C, overnight/–	
Clinoptilolite (Mud Hills (CA), USA)	Isotherm	5.90	25 °C, 1000 mbar	Zeolite	300 °C, overnight/–	
Faujasite dealuminated (Wessalith DAY F20)	Isotherm	1.77	25 °C, 1000 mbar	Zeolite	300 °C, overnight/–	
Pentasil dealuminated (Wessalith DAZ F20)	Isotherm	2.34	25 °C, 1000 mbar	Zeolite	300 °C, overnight/–	
Alumina (Compalox VPO2)	Isotherm	2.60	25 °C, 1000 mbar	Alumina	300 °C, overnight/–	
Alumina (LaRoche 1593)	Isotherm	2.15	25 °C, 1000 mbar	Alumina	300 °C, overnight/–	
Alumina (LaRoche 1597)	Isotherm	3.00	25 °C, 1000 mbar	Alumina	300 °C, overnight/–	
Silica gel 60 (Fluka 60742)	Isotherm	4.85	25 °C, 1000 mbar	Silica gel	200 °C, overnight/–	
Silica gel 100 (Fluka 60746)	Isotherm	3.60	25 °C, 1000 mbar	Silica gel	200 °C, overnight/–	
Silica gel 40 (Fluka 60736)	Isotherm	6.25	25 °C, 1000 mbar	Silica gel	200 °C overnight/–	
MCM‐41	Breakthrough	2.0	25 °C, 1.5 mbar	Mesoporous silica	120 °C, 8 h, vacuum/–	^[^ ^111^ ^]^
CoPBA	Breakthrough	1.9	25 °C, 0.01 mbar	Vacancy sites, interstitial sites	–/Water flushing	^[^ ^95^ ^]^
Prussian blue	Breakthrough	3.1	25 °C, 0.01 mbar	Vacancy sites, interstitial sites	–/Water flushing	
	Isotherm	12.5	25 °C, 1000 mbar	Vacancy sites, interstitial sites	100 °C, 24 h, vacuum/–	^[^ ^94^ ^]^
CoHCC	Isotherm	21.9	25 °C, 1000 mbar	Vacancy sites, interstitial sites	150 °C. 24 h, vacuum/–	
CuHCF	Isotherm	20.2	25 °C, 1000 mbar	Vacancy sites, interstitial sites	60 °C. 24 h, vacuum/–	
MOS‐1	Isotherm	11.5	25 °C, 1000 mbar	Metal–organic square	150 °C, overnight, vacuum/25 °C, 30 min, vacuum	^[^ ^91^ ^]^
MOS‐2	Isotherm	5.2	25 °C, 1000 mbar	Metal–organic square	150 °C, overnight, vacuum/25 °C, 30 min, vacuum	
MOS‐3	Isotherm	3.8	25 °C, 1000 mbar	Metal–organic square	150 °C, overnight, vacuum/25 °C, 30 min, vacuum	
Covalent organic frameworks						
COF‐10	Isotherm	15	25 °C, 1000 mbar	Lewis acidic boron sites	–/200 °C, 12 h, vacuum,	^[^ ^97^ ^]^
[HOOC]_0_‐COF	Isotherm	9.23	25 °C, 1000 mbar	Free carboxylic acid groups	180 °C, 24 h, vacuum/–	^[^ ^50^ ^]^
[HOOC]_17_‐COF	Isotherm	9.34	25 °C, 1000 mbar	Free carboxylic acid groups	180 °C, 24 h, vacuum/–	
[HOOC]_33_‐COF	Isotherm	8.21	25 °C, 1000 mbar	Free carboxylic acid groups	180 °C, 24 h, vacuum/–	
[HOOC]_50_‐COF	Isotherm	6.67	25 °C, 1000 mbar	Free carboxylic acid groups	180 °C, 24 h, vacuum/–	
[HOOC]_100_‐COF	Isotherm	4.14	25 °C, 1000 mbar	Free carboxylic acid groups	180 °C, 24 h, vacuum/–	
[CaOOC]_17_‐COF	Isotherm	12.25	25 °C,1000 mbar	Postsynthetic metalation	200 °C, 12 h, vacuum/–	
[MnOOC]_17_‐COF	Isotherm	11.38	25 °C, 1000 mbar	Postsynthetic metalation	200 °C, 12 h, vacuum/–	
[SrOOC]_17_‐COF	Isotherm	14.30	25 °C, 1000 mbar	Postsynthetic metalation	200 °C, 12 h, vacuum/200 °C, 12 h, vacuum	
Hydrogen‐bonded organic frameworks						
KUF‐1	Isotherm	6.67	25 °C, 1000 mbar	Cooperative adsorption due to structural transformation	120 °C, vacuum/RT, 10 h, vacuum	^[^ ^103^ ^]^
HOF‐102	Isotherm	0.11	25 °C, 1000 mbar	Large aromatic tectons	90 °C, overnight, vacuum/–	^[^ ^104^ ^]^
Porous organic polymers						
PAA	Isotherm	10.7	25 °C, 1000 mbar	Poly(amic acid)	<80 °C, N_2_/80 °C, 18 h	^[^ ^100^ ^]^
	Breakthrough	2.4	20 °C, 2.8 mbar, 0% RH		<80 °C, N_2_/–	
		4.4	20 °C, 2.8 mbar, 80% RH			
PI	Isotherm	9.0	25 °C, 1000 mbar	Polycyclic imide	<80 °C, N_2_/80 °C, 18 h	
	Breakthrough	1.1	20 °C, 2.8 mbar, 0% RH		<80 °C, N_2_/–	
		3.4	20 °C, 2.8 mbar, 80% RH			
1T	Isotherm	<3.8	25 °C, 1000 mbar	Free methyl groups	120 °C, 12 h, vacuum/–	^[^ ^108^ ^]^
1TC	Isotherm	6.41	25 °C, 1000 mbar	Postsynthetic incorporated free carboxylic acid groups	120 °C, 12 h, vacuum/–	
1TCS	Isotherm	8.52	25 °C, 1000 mbar	Postsynthetic incorporated free carboxylic and sulfonic acid groups	120 °C, 12 h, vacuum/–	
NU‐POP‐1	Breakthrough	5.56	20 °C, 1.44 mbar, 0% RH	Naphthalene diimide polymer	160 °C, 24 h/–	^[^ ^98^ ^]^
		6.17	20 °C, 1.44 mbar, 80% RH			
BPP‐5	Isotherm	17.7	25 °C, 1000 mbar	Postsynthetic incorporated free carboxylic acid groups	Appropriate temperature, vacuum/–	^[^ ^99^ ^]^
BPP‐7	Isotherm	16.1	25 °C, 1000 mbar	Postsynthetic incorporated free carboxylic acid groups	Appropriate temperature, vacuum/–	
P1‐PO_3_H_2_	Isotherm	18.7	25 °C, 1000 mbar	Postsynthetic incorporated free phosphonic acids	110 °C, vacuum/–	^[^ ^49^ ^]^
	Breakthrough	5.2	20 °C, 2.8 mbar, 0% RH		110 °C, vacuum/–	
		7.2	20 °C, 2.8 mbar, 80% RH			
P1‐NH_3_Cl	Isotherm	11.2	25 °C, 1000 mbar	Postsynthetic incorporated NH_3_Cl groups	110 °C, vacuum/–	
	Breakthrough	0.7	20 °C, 2.8 mbar, 0% RH		110 °C, vacuum/–	
		2.0	20 °C, 2.8 mbar, 80% RH			
P1‐SO_3_H	Isotherm	12.1	25 °C, 1000 mbar	Postsynthetic incorporated free sulfonic acid groups	120 °C, vacuum/–	
	Breakthrough	3.9	20 °C, 2.8 mbar, 0% RH		120 °C, vacuum/–	
		8.1	20 °C, 2.8 mbar, 80% RH			
P2‐NH_3_Cl	Isotherm	16.3	25 °C, 1000 mbar	Postsynthetic incorporated NH_3_Cl groups	100 °C, 24 h, vacuum/–	
	Breakthrough	1.0	20 °C, 2.8 mbar, 0% RH		100 °C, 24 h, vacuum/–	
		1.5	20 °C, 2.8 mbar, 80% RH			
P2‐CO_2_H	Isotherm	16.1	25 °C, 1000 mbar	Postsynthetic incorporated free carboxylic acid groups	110 °C vacuum/130 °C, 12 h, vacuum	
	Breakthrough	6.7	20 °C, 2.8 mbar, 0% RH		110 °C vacuum/–	
		7.4	20 °C, 2.8 mbar, 80% RH			
P2‐SO_3_H	Isotherm	13.1	25 °C, 1000 mbar	Postsynthetic incorporated free sulfonic acid groups	80 °C, vacuum/–	
	Breakthrough	4.0	20 °C, 2.8 mbar, 0% RH		80 °C, vacuum/–	
		4.3	20 °C, 2.8 mbar, 80% RH			
Other porous organic adsorbents						
Carboxen564	Breakthrough	0.04	25 °C, 1.2 mbar, 0% RH	Carbon molecular sieve	–/–	^[^ ^68^ ^]^
		0.11	25 °C, 1.2 mbar, 40% RH			
Carbosieve G	Breakthrough	0.58	25 °C, 1.2 mbar, 0% RH	Carbon molecular sieve	–/–	
		0.76	25 °C, 1.2 mbar, 40% RH			
BPL carbon	Breakthrough	0.58	25 °C, 9.9 mbar	–	–/–	^[^ ^59^ ^]^
BPL activated carbon	Breakthrough	0.25	25 °C, 0.45 mbar	–	150 °C, overnight, vacuum/–	^[^ ^113^ ^]^
AC	Breakthrough	0.02	25 °C, 0.01 mbar	Activated carbon	–/Water flushing	^[^ ^95^ ^]^
Activated carbon (Aldrich Darco 24226‐8)	Isotherm	4.19	25 °C, 1000 mbar	Activated carbon	200 °C, overnight/–	^[^ ^26^ ^]^
Activated carbon (Merck 1.09624)	Isotherm	5.08	25 °C, 1000 mbar	Activated carbon	200 °C, overnight/–	
Charcoal (Sigma C 3014)	Isotherm	5.27	25 °C, 1000 mbar	Carbon	200 °C, overnight/–	
C‐1	Breakthrough	0.55	25 °C, 1 mbar, 0% RH	Carbonized poly(4‐styrene sulfonic acid *co*‐maleic acid) sodium salt	120 °C, 24 h, air/–	^[^ ^126^ ^]^
		1.00	25 °C, 1 mbar, 70% RH			
C‐1A	Breakthrough	2.01	25 °C, 1 mbar, 0% RH	Oxidized with ammonium persulfate in sulfuric acid	120 °C, 24 h, air/–	
		2.74	25 °C, 1 mbar, 70% RH			
C‐1B	Breakthrough	0.66	25 °C, 1 mbar, 0% RH	Oxidized with ammonium persulfate in sulfuric acid	120 °C, 24 h, air/–	
		1.53	25 °C, 1 mbar, 70% RH			
C‐2	Breakthrough	1.01	25 °C, 1 mbar, 0% RH	Carbonized poly(sodium 4‐styrene sulfonate)	120 °C, 24 h, air/–	
		1.00	25 °C, 1 mbar, 70% RH			
C‐2A	Breakthrough	1.90	25 °C, 1 mbar, 0% RH	Oxidized with ammonium persulfate in sulfuric acid	120 °C, 24 h, air/–	
		2.23	25 °C, 1 mbar, 70% RH			
C‐2B	Breakthrough	0.63	25 °C, 1 mbar, 0% RH	Oxidized with ammonium persulfate in sulfuric acid	120 °C, 24 h, air/–	
		1.44	25 °C, 1 mbar, 70% RH			
12N N‐AC	Breakthrough	1.74	30 °C, 1 mbar	Acidified activated carbon	150 °C, 3 h, He/–	^[^ ^105^ ^]^
BAX	Breakthrough	0.38	25 °C, 1 mbar, 0% RH	Activated carbons modified with aluminum–zirconium polycations	120 °C/–	^[^ ^115^ ^]^
		0.48	25 °C, 1 mbar, 70% RH			
BAX‐300	Breakthrough	0.76	25 °C, 1 mbar, 0% RH	Activated carbons modified with aluminum–zirconium polycations	120 °C/–	
		1.16	25 °C, 1 mbar, 70% RH			
BAX‐R	Breakthrough	0.85	25 °C, 1 mbar, 0% RH	Activated carbons modified with aluminum–zirconium polycations	120 °C/–	
		0.87	25 °C, 1 mbar, 70% RH			
BAX‐R300	Breakthrough	0.53	25 °C, 1 mbar, 0% RH	Activated carbons modified with aluminum–zirconium polycations	120 °C/–	
		0.94	25 °C, 1 mbar, 70% RH			
NPC‐PEF‐AC‐F	Isotherm	17	25 °C, 1000 mbar	Polyfurfuryl alcohol derived carbon acidified with nitric acid	90 °C, 12 h, vacuum/vacuum	^[^ ^106^ ^]^
Fe_3_C‐CDC‐600C‐0.75 h	Breakthrough	1.88	25 °C, 1.5 mbar, 0% RH	Chlorinated Fe_3_C carbide	150 °C, 2 h, N_2_/–	^[^ ^107^ ^]^
		3.44	25 °C, 1.5 mbar, 75% RH			
Fe_3_C‐CDC‐600C‐1 h	Breakthrough	1.75	25 °C, 1.5 mbar, 0% RH	Chlorinated Fe_3_C carbide	150 °C, 2 h, N_2_/–	
		2.45	25 °C, 1.5 mbar, 75% RH			
Fe_3_C‐CDC‐600C‐1.5 h	Breakthrough	1.62	25 °C, 1.5 mbar, 0% RH	Chlorinated Fe_3_C carbide	150 °C, 2 h, N_2_/–	
		2.91	25 °C, 1.5 mbar, 75% RH			
Fe_3_C‐CDC‐600C‐5 h	Breakthrough	0.17	25 °C, 1.5 mbar, 0% RH	Chlorinated Fe_3_C carbide	150 °C, 2 h, N_2_/–	
		1.01	25 °C, 1.5 mbar, 75% RH			
GO‐ED	Breakthrough	1.57	25 °C, 1 mbar	Graphite oxide	120 °C, overnight, air/–	^[^ ^123^ ^]^
GO‐EM	Breakthrough	1.82	25 °C, 1 mbar	Graphite oxide	120 °C, overnight, air/–	
GO1	Breakthrough	2.87	25 °C, 1 mbar, 0% RH	Graphite oxide	120 °C, vacuum/–	^[^ ^124^ ^]^
		2.11	25 °C, 1 mbar, 70% RH			
GOB	Breakthrough	0.41	25 °C, 1 mbar, 0% RH	Graphite oxide	120 °C, vacuum/–	
		1.05	25 °C, 1 mbar, 70% RH			
GO	Breakthrough	3.25	25 °C, 1 mbar, 0% RH	Graphite oxide	–/–	^[^ ^117^ ^]^
		3.58	25 °C, 1 mbar, 70% RH			
		2.09	1 mbar, 0% RH		120 °C, vacuum/–	^[^ ^116^ ^]^
		3.29	1 mbar, humid			
		3.24	1 mbar, 70% RH			
		2.64	25 °C, 1 mbar, 0% RH		–/–	^[^ ^119^ ^]^
		1.93	25 °C, 1 mbar, 0% RH			
		2.6	25 °C, 1 mbar		–/–	^[^ ^121^ ^]^
GO‐E	Breakthrough	3.28	1 mbar	Graphite oxide	130–135 °C, 6 h, vacuum/–	^[^ ^118^ ^]^
IE resin	Breakthrough	0.38	25 °C, 0.01 mbar	Ion exchange resin	–/Water flushing	^[^ ^95^ ^]^
Polymer resin (Macronet (MN) 200)	Isotherm	5.20	25 °C, 1000 mbar	Polymer	115 °C, overnight/–	^[^ ^26^ ^]^
Polymer resin (Amberlyst 15)	Isotherm	11.34	25 °C, 1000 mbar	Polymer	115 °C, overnight/–	
Composites						
30‐HKUST‐1 MMM	Breakthrough	1.3	20 °C, 2.88 mbar	HKUST‐1/PVDF composite	100 °C, 1 h, air/–	^[^ ^112^ ^]^
50‐HKUST‐1 MMM	Breakthrough	3.2	20 °C, 2.88 mbar	HKUST‐1/PVDF composite	100 °C, 1 h, air/–	
67‐HKUST‐1 MMM	Breakthrough	4.9	20 °C, 2.88 mbar	HKUST‐1/PVDF composite	100 °C, 1 h, air/–	
Cu‐MCM‐BTC	Breakthrough	5.2	25 °C, 1.5 mbar	HKUST‐1/mesoporous silica composite	120 °C, 8 h, vacuum/–	^[^ ^111^ ^]^
10GO1M	Breakthrough	5.63	25 °C, 1 mbar, 0% RH	HKUST‐1/GO composite	120 °C, vacuum/–	^[^ ^124^ ^]^
		7.04	25 °C, 1 mbar, 70% RH			
30GO1M	Breakthrough	2.52	25 °C, 1 mbar, 0% RH	HKUST‐1/GO composite	120 °C, vacuum/–	
		7.33	25 °C, 1 mbar, 70% RH			
10GOBM	Breakthrough	4.11	25 °C, 1 mbar, 0% RH	HKUST‐1/GO composite	120 °C, vacuum/–	
		4.93	25 °C, 1 mbar, 70% RH			
30GOBM	Breakthrough	2.46	25 °C, 1 mbar, 0% RH	HKUST‐1/GO composite	120 °C, vacuum/–	
		5.57	25 °C, 1 mbar, 70% RH			
MG‐1	Breakthrough	7.51	25 °C, 1 mbar, 0%	HKUST‐1/GO composite	–/–	^[^ ^119^ ^]^
		11.74	25 °C, 1 mbar, humid			
MG‐2	Breakthrough	7.61	25 °C, 1 mbar, 0%	HKUST‐1/GO composite	–/–	
		13.56	25 °C, 1 mbar, humid			
MG‐3	Breakthrough	8.74	25 °C, 1 mbar, 0%	HKUST‐1/GO composite	–/–	
		10.68	25 °C, 1 mbar, humid			
MG‐4	Breakthrough	5.10	25 °C, 1 mbar, 0%	HKUST‐1/GO composite	–/–	
		8.63	25 °C, 1 mbar, humid			
MG‐5	Breakthrough	4.11	25 °C, 1 mbar, 0%	HKUST‐1/GO composite	–/–	
		7.22	25 °C, 1 mbar, humid			
MGO1	Isotherm	13.2	28 °C, 1200 mbar	HKUST‐1/GO composite	120 °C, 6 h, vacuum/–	^[^ ^121^ ^]^
	Breakthrough	7.5	25 °C, 1 mbar		–/–	
MGO2	Isotherm	14.5	28 °C, 1200 mbar	HKUST‐1/GO composite	120 °C, 6 h, vacuum/–	
	Breakthrough	77	25 °C, 1 mbar		–/–	
MGO3	Isotherm	11.6	28 °C, 1200 mbar	HKUST‐1/GO composite	120 °C, 6 h, vacuum/–	
	Breakthrough	8.8	25 °C, 1 mbar		–/–	
HKUST‐1/GO	Isotherm	≈5.4	40 °C, 1000 mbar	HKUST‐1/GO composite	120 °C, 1.5 h, vacuum/120 °C, vacuum	^[^ ^122^ ^]^
CuMG‐1	Breakthrough	11.74	25 °C, 1 mbar, humid	HKUST‐1/GO composite	120 °C/–	^[^ ^120^ ^]^
CuMG‐2	Breakthrough	10.68	25 °C, 1 mbar, humid	HKUST‐1/GO composite	120 °C/–	
ZnMG‐1	Breakthrough	3.11	25 °C, 1 mbar, humid	MOF‐5/GO composite	120 °C/–	
ZnMG‐2	Breakthrough	4.69	25 °C, 1 mbar, humid	MOF‐5/GO composite	120 °C/–	
MOF‐5/GO1‐E	Breakthrough	0.41	1 mbar	MOF‐5/GO composite	–/–	^[^ ^118^ ^]^
MOF‐5/GO2‐E	Breakthrough	1.29	1 mbar	MOF‐5/GO composite	–/–	
MOF‐5/GO3‐E	Breakthrough	2.23	1 mbar	MOF‐5/GO composite	–/–	
MOF‐5/GO4‐E	Breakthrough	4.81	1 mbar	MOF‐5/GO composite	–/–	
MOF‐5–GO	Breakthrough	0.40	25 °C, 1 mbar, 0% RH	MOF‐5/GO composite	–/–	^[^ ^117^ ^]^
		3.12	25 °C, 1 mbar, 70% RH			
MIL‐GO1‐ED	Breakthrough	2.85	25 °C, 1 mbar	MIL‐100(Fe)/GO composite	120 °C, overnight, air/–	^[^ ^123^ ^]^
MIL‐GO1‐EM	Breakthrough	2.59	25 °C, 1 mbar	MIL‐100(Fe)/GO composite	120 °C, overnight, air/–	
MIL‐GO1d‐ED	Breakthrough	5.31	25 °C, 1 mbar	MIL‐100(Fe)/GO composite	120 °C, overnight, air/–	
MIL‐GO2‐ED	Breakthrough	2.58	25 °C, 1 mbar	MIL‐100(Fe)/GO composite	120 °C, overnight, air/–	
MIL‐GO2‐EM	Breakthrough	1.66	25 °C, 1 mbar	MIL‐100(Fe)/GO composite	120 °C, overnight, air/–	
MIL‐GO2d‐ED	Breakthrough	3.54	25 °C, 1 mbar	MIL‐100(Fe)/GO composite	120 °C, overnight, air/–	
STAM‐17‐OEt@BPL_1	Breakthrough	1.04	25 °C, 0.45 mbar	STAM‐17‐OEt/activated carbon composite	150 °C, overnight, vacuum/–	^[^ ^113^ ^]^
STAM‐17‐OEt@BPL_2	Breakthrough	0.83	25 °C, 0.45 mbar	STAM‐17‐OEt/activated carbon composite	150 °C, overnight, vacuum/–	
STAM‐17‐OEt@BPL_3	Breakthrough	0.78	25 °C, 0.45 mbar	STAM‐17‐OEt/activated carbon composite	150 °C, overnight, vacuum/–	
GO‐W	Breakthrough	2.53 / /	1 mbar, 0% RH	GO/polyoxometalate composite	120 °C, vacuum/–	^[^ ^116^ ^]^
		2.01	1 mbar, humid			
		4.06	1 mbar, 70% RH			
GO‐Mo	Breakthrough	3.38	1 mbar, 0% RH	GO/polyoxometalate composite	120 °C, vacuum/–	
		3.52	1 mbar, humid			
		4.37	1 mbar, 70% RH			

## 2 Porous Inorganic–Organic Hybrid Adsorbents

Studies involving porous inorganic–organic hybrid NH_3_ adsorbents have been reported; such studies were mainly focused on MOFs that are constructed by metal clusters and organic linkers, owing to the advantages of such MOFs for gas capture. Promising strategies that employ MOFs for NH_3_ adsorption are the formation of open‐metal sites or the postsynthetic functionalization of organic ligands in the framework. Open‐metal sites can be developed by removing the solvent molecules coordinated to a metal cluster, commonly observed in robust frameworks such as the MOF‐74 series. Particularly, sites with strong Lewis acidity capture basic NH_3_ gas even at extremely low pressures. By contrast, organic linkers are manipulated by donating further acidic sites to change the chemical environments in the pores. This is a useful method to promote NH_3_ removal because the decorated functional groups enhance the affinity of NH_3_ toward the framework. A few hybrid materials such as metal–organic square and Prussian blue have also been reported as effective NH_3_ adsorbents.

### 2.1 Metal–Organic Frameworks

MOFs, defined as materials composed of metal ions (or clusters) and organic spacers, have numerous advantages such as a large surface area and pore volume, tunable functionality, and structural versatility. To effectively remove NH_3_, the structural stability of a framework is vital because the high basicity of NH_3_ disrupts the crystalline structure of the framework. Thus, robust MOFs such as MOF‐74, UiO‐66, and MIL‐101, which possess high stability under harsh conditions, have been utilized in the removal of harmful gases, including NH_3_.

**Figure 2:**
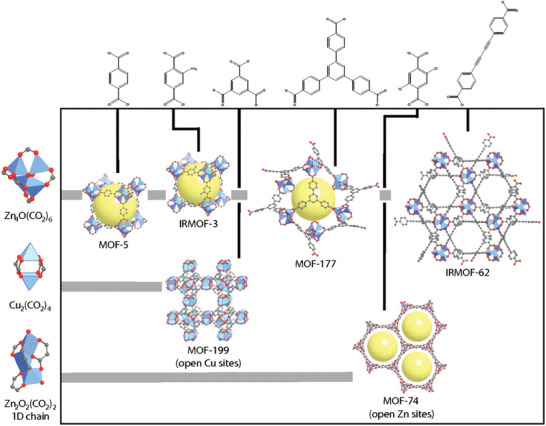
Crystal structures of MOF‐5, IRMOF‐3, MOF‐177, IRMOF‐62, and MOF‐199. (C: gray; O: red; N: teal; metal ions: blue polyhedra; H atoms were removed for clarity). Reproduced with permission.^[^ ^59^ ^]^ Copyright 2008, National Academy of Sciences.

Substantial research has been focused on the MOF‐74 system due to the existence of Lewis acidic metal centers that can act as interaction sites with the basic NH_3_. Yaghi and co‐workers studied the gas capacity and selectivity of six MOFs (MOF‐5, IRMOF‐3, MOF‐74, MOF‐177, MOF‐199, and IRMOF‐62) for harmful gases including NH_3_ (**Figure** **2**).^[^ ^59^ ^]^ Through kinetic breakthrough measurements, the dynamic adsorption capacity of each MOF was determined under 0.99% NH_3_ with the N_2_ balance at 25 mL min^−1^.

The performance of the MOFs was compared with that of Calgon BPL activated carbon, which is widely used in industry. The amounts of NH_3_ adsorbed on the MOFs and carbon were 0.006 (MOF‐5), 0.105 (IRMOF‐3), 0.093 (MOF‐74), 0.042 (MOF‐177), 0.087 (MOF‐199), 0.023 (IRMOF‐62), and 0.001 (BPL carbon) g g^−1^. In contrast with MOF‐5, the existence of amines in IRMOF‐3 significantly affected the adsorption of NH_3_ owing to additional hydrogen bonds. Furthermore, the uptake amount of IRMOF‐3 was 71 times greater than that of BPL carbon. This work revealed the excellent potential of MOFs as NH_3_ adsorbents.

Similarly, Saha and Deng investigated the stability of the frameworks (MOF‐5 and MOF‐177) before and after NH_3_ adsorption.^[^ ^60^ ^]^ Before NH_3_ adsorption, the BET (Brunauer–Emmett–Teller) surface areas of MOF‐5 and MOF‐177 were 2449 and 3275 m^2^ g^−1^; after NH_3_ adsorption, the areas substantially decreased to 10 and 4 m^2^ g^−1^, respectively. In addition, the crystallinity of the MOFs totally vanished. Fourier transform infrared (FT‐IR) and Raman spectra suggested the existence of free organic ligands in each MOF, indicating the destruction of the frameworks.

**Figure 3:**
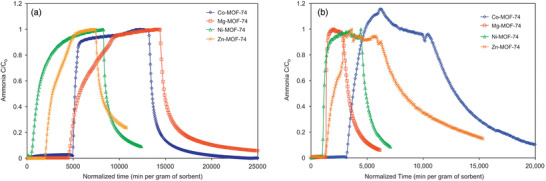
NH_3_ breakthrough curves of MOF‐74 analogs in a stream of 1000 mg m^−3^ NH_3_ at 25 °C under a) dry (0% RH) and b) humid (80% RH) conditions with a flow rate of 20 mL min^−1^. Desorption is proceeded by passing clean air under same conditions. Reproduced with permission.^[^ ^61^ ^]^ Copyright 2011, Elsevier.

The ability of MOF‐74 analogs, M‐MOF‐74 (M = Zn, Co, Ni, Mg), to remove toxic gases from air, including NH_3_, was inspected by Glover et al. (**Figure** **3**).^[^ ^61^ ^]^ Such MOFs retain the 1D chain structure of M_2_O_2_(CO_2_)_2_ (M = Zn, Co, Ni, Mg), connected by 2,5‐dioxidoterephthalate. The gas adsorption capacity of the MOFs was determined by fixed‐bed microbreakthrough measurements under dry and humid gas streams. In the dry condition (0% relative humidity (RH)), Mg‐MOF‐74 exhibited the highest NH_3_ loading of 7.60 mol kg^−1^, followed by Co‐MOF‐74 with 6.70 mol kg^−1^, which greatly surpasses the NH_3_ loading of traditional porous adsorbents, such as BPL activated carbon (0.17 mol kg^−1^) and 13X zeolite (2.89 mol kg^−1^). Furthermore, the Mg and Co analogues retained significant amounts of adsorbed NH_3_ (70% and 83%, respectively) during the desorption process, in contrast to the nearly 0% retention of NH_3_ by 13X zeolite. Although the NH_3_ uptake of all analogues decreased in humid conditions (80% RH), the loading still exceeded that of the carbon and zeolite. The high affinity of the MOFs for NH_3_ and the weak correlation between the NH_3_ uptake and the calculated BET surface area suggest that the adsorption capabilities are more dependent on the adsorbent–adsorbate interaction than the surface area. The study highlighted the potential for the use of MOFs as NH_3_ adsorbents under dry and humid conditions.

To test the stability of MOFs upon NH_3_ adsorption, Kitagawa and co‐workers investigated 16 known MOFs by comparing powder X‐ray diffraction (PXRD) patterns before and after exposure to NH_3_ for 2 h at different temperatures.^[^ ^62^ ^]^ Candidates were selected to examine the influence of metal cations and organic linkers on the stability of the structure. Among the tested materials, MIL‐53 (Al), Al‐BTB, MOF‐76 (M) (M = Y or Yb), MIL‐101(Cr), MOF‐74(Mg), and ZIF‐8 maintained their structures at temperatures up to 350 °C under an NH_3_ atmosphere at 1 atm. Generally, MIL‐53(Al), Al‐BTB, MOF‐76(Y), MOF‐76(Yb), and MIL‐101(Cr) with oxophilic M^III^ centers and oxygen donors exhibited high stability against NH_3_. Moreover, the frameworks possessing chemically inert M^III^ were less reactive with NH_3_. Although most frameworks with divalent central metal cations decomposed when in contact with NH_3_, the strong coordination of *ortho*‐positioned oxygen atoms in the dobdc (dobdc^4−^ = 2,5‐dioxido‐1,4‐benzenedicarboxylate) linker forming a chelate structure with Mg^II^ appeared to contribute to the high stability of MOF‐74. With regard to ZIF‐8, the strong metal‐linker bond associated with the anionic N donor (2‐methylimidazolate) accounts for the high stability, in contrast to the weak stability of MOFs with neutral nitrogen donors (4,4′‐bipyridine). Notably, most of the MOFs that were stable against NH_3_ were also reported to be stable under humid conditions. The NH_3_ adsorption isotherm measurements revealed the distinct adsorptive behavior of the robust MOFs. This result revealed the diverse potential applications of such MOFs, including their application as NH_3_ sorbents, Lewis acid catalysts, and as supports for NH_3_ synthesis or decomposition.

**Figure 4:**
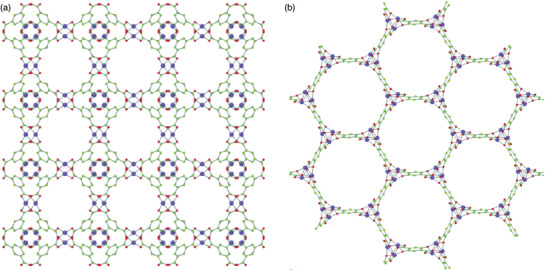
3D structures. a) Cubic HKUST‐1 and b) honeycomb Cu‐MOF‐74 with the open metal sites of Cu(II). Reproduced with permission.^[^ ^64^ ^]^ Copyright 2016, The Royal Society of Chemistry.

Previously, the Lewis‐acid/base coordination of NH_3_ molecules with the open sites on the Cu(II) centers of HKUST‐1 or Cu_3_(BTC)_2_ (BTC = 1,3,5‐benzenetricarboxylate) was revealed to account for the outstanding NH_3_ uptake.^[^ ^63^ ^]^ Farha and co‐workers reported the maximum volumetric NH_3_ uptake of Cu‐MOF‐74 under humid conditions (**Figure** **4**).^[^ ^64^ ^]^ Cu‐MOF‐74 maintained the highest density of Cu(II) sites per unit volume (4.7 nm^−3^) among all of the MOFs, including HKUST‐1 (2.2 nm^−3^). Interestingly, breakthrough NH_3_ measurements revealed that increasing the humidity from 0% to 80% RH more than doubles the adsorption from 3.4 to 7.6 mmol g^−1^ or 0.56 molecules per Cu(II) center to 1.2 molecules per Cu(II) center. In volumetric terms, the NH_3_ uptake at 80% RH translated to the highest uptake of 5.9 NH_3_ nm^−3^, far exceeding that of 3.9 NH_3_ nm^−3^ for HKUST‐1. However, PXRD conducted after the adsorption measurement under humid conditions indicated a loss of crystallinity. This is because the breaking of Cu‐carboxylate bonds presumably leads to the formation of (NH_4_)_3_BTC and Cu(OH)_2_ species upon exposure of Cu‐MOF‐74 to NH_3_. Despite this structural degradation, the high NH_3_ capacity makes Cu‐MOF‐74 a promising material for use in single‐use filters or abatement systems.

**Figure 5:**
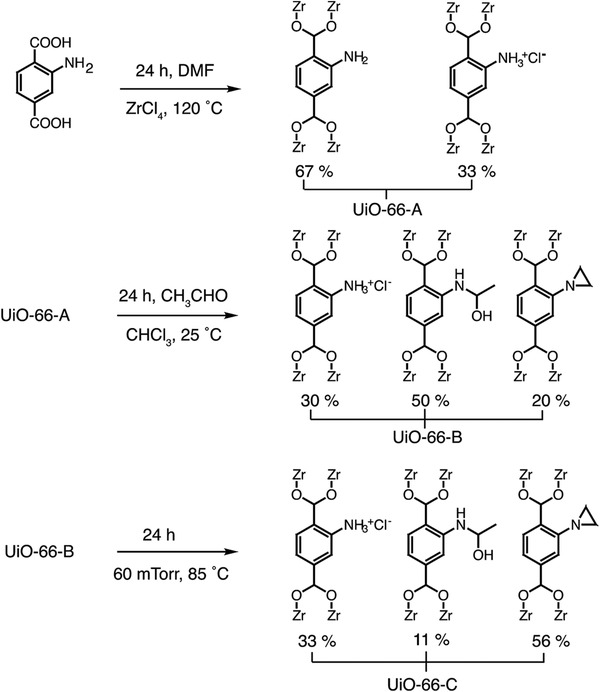
Preparation and postsynthetic modifications of UiO‐66‐A. Reproduced with permission.^[^ ^65^ ^]^ Copyright 2011, American Chemical Society.

UiO‐66 and its various derivatives have also received substantial attention as porous platforms for NH_3_ removal owing to their robust frameworks and tunable functionalities. Yaghi and co‐workers synthesized UiO‐66‐A [Zr_6_O_4_(OH)_4_(BDC‐NH_2_)_4_(BDC‐NH_3_^+^Cl^−^)_2_] (BDC = 1,4‐benzenedicarboxylate), which was composed of amino‐ and NH_3_^+^Cl^−^‐functionalized BDC mixed linkers and Zr‐clusters (**Figure** **5**).^[^ ^65^ ^]^ Then, UiO‐66‐A was reacted with acetaldehyde in CHCl_3_ to produce UiO‐66‐B with hemiaminal functional groups as the major species. After heat treatment under vacuum at 85 °C for 24 h, UiO‐66‐C with aziridine was obtained. From the ^15^N NMR data, the proportion of protonated amine/hemiaminal/aziridine was determined to be 3:5:2 for UiO‐66‐B and 3:1:5 for UiO‐66‐C. From N_2_ adsorption measurements at 77 K, BET surface areas were determined to be 820 m^2^ g^−1^ (UiO‐66‐A), 780 m^2^ g^−1^ (UiO‐66‐B), and 800 m^2^ g^−1^ (UiO‐66‐C). Interestingly, modified MOFs (UiO‐66‐B and UiO‐66‐C) showed NH_3_ capacities of 159 and 193 cm^3^ g^−1^, respectively, which were superior to the amount adsorbed (159 cm^3^ g^−1^) of UiO‐66‐A at 760 Torr.

**Figure 6:**
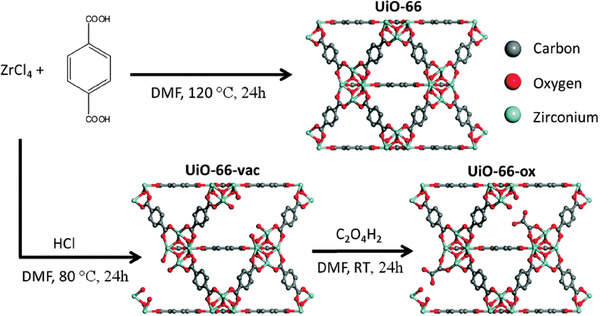
Synthetic procedure of UiO‐66, UiO‐66‐vac, and UiO‐66‐ox. Reprinted with permission.^[^ ^66^ ^]^ Copyright 2015, The Royal Society of Chemistry.

In the study by Farha and co‐workers, using a previously reported method, UiO‐66‐vac was prepared via the reaction of ZrCl_4_ and benzene‐1,4‐dicarboxylic acid in DMF with HCl (**Figure** **6**).^[^ ^66^ ^]^ MOFs have vacant sites where terephthalic linkers are not incorporated; these sites were reacted with oxalic acid solution to produce UiO‐66‐ox with retention of the same framework structure. Moreover, from the NMR data, the ratio of oxalic acid to terephthalic acid was ≈0.3. From the NH_3_ microbreakthrough measurements, the amounts of NH_3_ adsorbed by the MOFs were 2.0 mmol g^−1^ for UiO‐66, 1.6 mmol g^−1^ for UiO‐66‐vac, and 2.5 mmol g^−1^ for UiO‐66‐ox. The increased NH_3_ uptake of UiO‐66‐ox can be explained by two factors. One is the free carboxylic acids in the framework, which forms an ammonium carboxylate species with NH_3_, and the other is the binding of NH_3_ to carboxylic acid through hydrogen bonds. This work suggested that the defect in the framework can be positively utilized in NH_3_ capture via postsynthetic modification.

Likewise, Walton and co‐workers solvothermally synthesized Zr‐based UiO‐66 analogues (UiO‐66‐OH, UiO‐66‐(OH)_2_, UiO‐66‐NO_2_, UiO‐66‐NH_2_, UiO‐66‐SO_3_H, and UiO‐66‐(COOH)_2_) and investigated their NH_3_ adsorption performance with breakthrough measurements in dry (0% RH) and humid (80% RH) conditions.^[^ ^67^ ^]^ Counterintuitively, UiO‐66‐OH exhibited the highest NH_3_ capacity of ≈5.7 mmol g^−1^, followed by UiO‐66‐NH_2_ (≈3.6 mmol g^−1^) in dry conditions, outperforming frameworks with more acidic functional groups, namely, UiO‐66‐SO_3_H and UiO‐66‐(COOH)_2_ (<3 mmol g^−1^). This indicates that the inaccessible pores arising from the reduced surface area and the pore volume obtained upon grafting bulky functional groups to UiO‐66 could lead to a decreased NH_3_ capture ability. However, the NH_3_ capacity decreased in all UiO‐66 variants in humid conditions, in contrast with the parent framework, possibly owing to the more hydrophilic nature of the variants, which promotes competition between H_2_O and NH_3_ on the functionalized active sites. This work revealed that NH_3_ capture relies on the interplay of various factors, including the functional group and porosity.

Several porous materials, such as MOFs (UiO‐66‐NH_2_, UiO‐66‐COOH, UiO‐66‐fumarate, Ni‐MOF‐74, Zn‐MOF‐74, Fe‐BTC, Cu‐BTC, and Al‐MIL‐101‐NH_2_), zeolites (ZSM‐5, Beta, and faujasites), and carbon molecular sieves (Carboxen 564 and Carbosieve G 60/80), were studied by Khabzina and Farrusseng for NH_3_ capture to unveil the mechanisms of NH_3_ adsorption under humid conditions.^[^ ^68^ ^]^ The NH_3_ capacities of the adsorbents were evaluated via breakthrough measurements under a 100 mL min^−1^ gas stream with 1200 ppm NH_3_ in dry or humid (40% RH) conditions. The NH_3_ adsorption mechanism in microporous solids can be explained via solubilization, physisorption, and chemisorption. In the case of the MOF adsorbents, humidity can either positively or negatively affect the NH_3_ uptake, depending on the type of each MOF. This is because diverse NH_3_ adsorption mechanisms co‐occur with respect to the characteristics of the adsorbents. However, it is undeniable that the role of humidity is crucial in all cases. In particular, in humid conditions, NH_3_ adsorption follows Henry's law, indicating an NH_3_ solubilization‐like mechanism. This mechanism dominates when the relative humidity surpasses the alpha value (the pivotal value of relative humidity at which half of the micropore volume is occupied). Furthermore, NH_3_ adsorption is significantly related to the number of condensed H_2_O molecules in the micropores of the solid with the exception of Cu‐MOFs, for which chemisorption with NH_3_ is facilitated.

**Figure 7:**
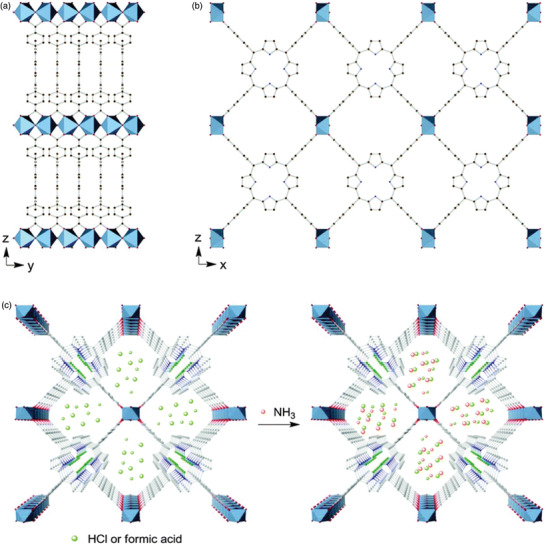
Crystal structure of Al‐PMOF along the a) [100] and b) [010] directions. c) Schematic of acid‐loaded Al‐PMOF for the capture of NH_3_. Reproduced with permission.^[^ ^69^ ^]^ Copyright 2015, The Royal Society of Chemistry.

Acid‐impregnated porphyrin‐based MOFs for NH_3_ capture were reported by Rosseinsky and co‐workers.^[^ ^69^ ^]^ They prepared Al_2_(OH)_2_(H_2_TCPP) (Al‐PMOF, H_2_TCPP = *meso*‐tetra(4‐carboxyl‐phenyl) porphyrin) based on infinite Al(OH)O_4_ chains and free‐base porphyrin linkers (**Figure** **7**a). After the evacuation of the pores in the framework, HCl and formic acid were introduced to the pores to produce Al‐PMOF‐HCl and Al‐PMOF‐FA, respectively (Figure 7b). After acid impregnation, the crystallinity of both frameworks was maintained. To evaluate the NH_3_ uptake of the MOFs, kinetic microbreakthrough measurements at 500 ppm of NH_3_ were conducted under dry and humid (80% RH) conditions. Intriguingly, acid‐impregnated MOFs exhibited a longer breakthrough time and higher NH_3_ uptake than those of the parent MOFs. Specifically, the time taken and the amounts of NH_3_ adsorbed by Al‐PMOF‐HCl were 226 min and 7.9 wt% under humid conditions, respectively, which is superior to the performance of Al‐PMOF (25 min and 0.9 wt%) at the same conditions. The PXRD results revealed that the crystallinity of Al‐PMOF‐HCl considerably decreased after the breakthrough measurements. An additional peak was observed in the XRD pattern, indicating the formation of ammonium chloride.

**Figure 8:**
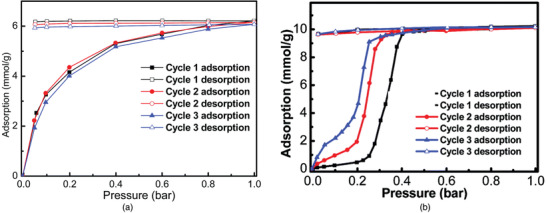
NH_3_ recyclability isotherm curves of a) Zn(INA)_2_ and b) Zn(NA)_2_ at 25 °C. Reproduced with permission.^[^ ^70^ ^]^ Copyright 2016 , The Royal Society of Chemistry. Reproduced with permission.^[^ ^71^ ^]^ Copyright 2018, The Royal Society of Chemistry.

In addition to the MOF‐74 and UiO‐66 series, candidate MOFs prepared with a combination of different metal ions and organic linkers were examined as potential NH_3_ adsorbents. Li and co‐workers studied NH_3_ adsorption properties using Zn(INA)_2_(H_2_O)_4_ (INA = isonicotinate).^[^ ^70^ ^]^ Dehydrated Zn(INA)_2_ was obtained from the activation of the original MOF, and structural crystallinity was confirmed by PXRD patterns. At 1 bar, the amount of NH_3_ adsorbed by Zn‐MOF was recorded to be 6 mmol g^−1^, and this amount was sustained after even 3 adsorption–desorption cycles (**Figure** **8**a). The stability of Zn‐MOF was investigated using an NH_3_ solution to generate the NH_3_+H_2_O covapor. Under covapor environments, the PXRD peaks of the MOF shifted. The changed structure was designated as Zn(INA)_2_(H_2_O)_2_(NH_3_)_2_. The vapor‐adsorbed MOF was transformed to Zn(INA)_2_ through the removal of solvents at 150 °C. This work presented a Zn‐MOF with high recyclability prepared via a new synthetic method using NH_3_ vapor diffusion.

The NH_3_ capacities of flexible M(NA)_2_ (M = Zn, Co, Cu, and Cd; NA = nicotinate) were reported by Li and co‐workers^[^ ^71^ ^]^ Although synthetic methods for 2D MOFs, Zn(NA)_2_ and Co(NA)_2_, and 3D MOFs, Cu(NA)_2_ and Cd(NA)_2_, were reported previously, the authors prepared the MOFs through NH_3_‐assisted synthesis or the solvent‐evaporation conversion method.^[^ ^71^, ^72^, ^73^, ^74^ ^]^ To investigate the NH_3_ uptake of the MOFs, NH_3_ adsorption–desorption measurements were conducted for 3 cycles. In the case of Zn(NA)_2_, the amount adsorbed slightly increased below 0.22 bar, and then sharply increased to 10.2 mmol g^−1^ at 0.4 bar (Figure 8b). This adsorption behavior can be explained by an ordinary gate‐opening phenomenon, which is due to the characteristics of the layered structure of Zn(NA)_2_.^[^ ^75^, ^76^ ^]^ After the adsorption measurements, the Zn(NA)_2_ peaks changed or shifted in the PXRD patterns, indicating the opening of the layered space and enlargement of the pores. Furthermore, the amount adsorbed by Co(NA)_2_ reached 17.5 mmol g^−1^, one of the highest reported values, but the crystallinity of this MOF completely vanished despite its reusable performance for 3 cycles. This work demonstrated that the flexibility in a MOF can affect the uptake and reusable performance with regard to NH_3_ capture.

**Figure 9:**
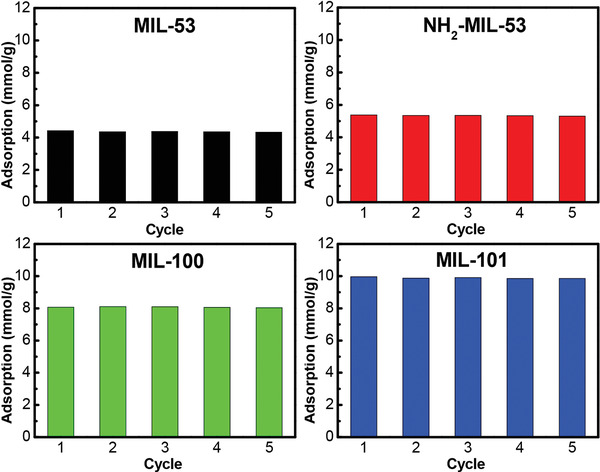
NH_3_ adsorption capacities of the MIL‐MOF series for repeated isotherm cycles at 298 K. Reproduced with permission.^[^ ^36^ ^]^ Copyright 2018, Elsevier.

MIL‐MOFs are widely known as useful materials with high structural stability. A family of MIL (MIL stands for Materials of Institute Lavoisier) was investigated by Yang and co‐workers, in respect of NH_3_ uptake ^[^ ^36^ ^]^ The MOFs are connected by trivalent metals and terephthalic acid or its derivatives. MIL‐53 (Al), NH_2_‐MIL‐53 (Al), MIL‐100 (Al), and MIL‐101 (Cr) were synthesized according to reported procedures.^[^ ^77^, ^78^, ^79^, ^80^ ^]^ According to the NH_3_ isotherms of the MOFs at 298 K, MIL‐101 showed the highest NH_3_ capacity (10 mmol g^−1^) at 1 bar among the MIL‐series (4.4 mmol g^−1^ for MIL‐53 and 8 mmol g^−1^ for NH_2_‐MIL‐53) due to the largest surface area. At low pressures (<0.1 bar), the NH_3_ uptake of NH_2_‐MIL‐53 was superior to that of MIL‐53, which was ascribed to the presence of the amino groups that increased the number of NH_3_ adsorption sites. Additionally, owing to the effect of the amine groups in NH_2_‐MIL‐53, the desorption curve of the MOF exhibited lagging because of strong adsorption. The adsorption performances of the MOFs were maintained for 5 cycles because of their robust frameworks (**Figure** **9**).

**Figure 10:**
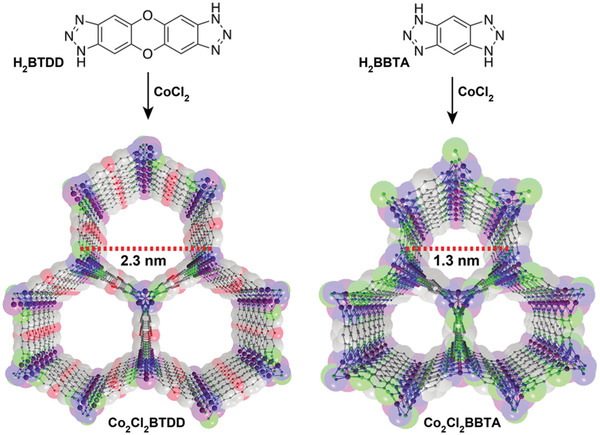
Crystal structure of Co_2_Cl_2_BTDD (left) and Co_2_Cl_2_(BBTA) (C: gray; O: red; N: blue; Cl: green; Co: purple; H atoms were removed for clarity). Reproduced with permission.^[^ ^82^ ^]^ Copyright 2018, American Chemical Society.

Dincă and co‐workers studied several new mesoporous MOFs, Mn_2_Cl_2_(BTDD)(H_2_O)_2_, Co_2_Cl_2_(BTDD)(H_2_O)_2_, and Ni_2_Cl_2_(BTDD)(H_2_O)_2_, composed of bis(1*H*‐1,2,3,‐triazolo[4,5‐*b*],[4′,5′‐*i*])dibenzo[1,4]dioxin (H_2_BTDD), which is an extended version of 1*H*,5*H*‐benzo(1,2,‐*d*:4,5‐*d*′)bistriazole (H_2_BBTA) (**Figure** **10**).^[^ ^81^ ^]^ The crystal structure of Mn‐MOF, which was elucidated via single‐crystal X‐ray diffraction, showed a honeycomb arrangement along the *c*‐axis with ≈22 Å wide mesoporous channels. Two chlorides contributed to the framework as bridging ligands. The meso‐porosities of the activated MOFs were surveyed using the N_2_ isotherm at 77 K, which exhibited a type‐IV pattern. The BET surface area of each sample was calculated to be 1917 (Mn), 1912 (Co), and 1752 m^2^ g^−1^ (Ni). In the NH_3_ adsorption isotherms at 298 K, the samples displayed adsorbed amounts of 15.47 (Mn), 12.00 (Co), and 12.02 mmol g^−1^ (Ni), which were in the high range among those of the porous materials. The high uptake values originating from the open metal sites showed the strong Lewis acidity of each framework. Particularly, owing to such sites, the Mn‐MOF exhibited an NH_3_ uptake of 9.35 mmol g^−1^ at the lowest pressure point collected during the desorption process. Additionally, the NH_3_ uptake performances of the MOFs were maintained under repeated measurements for at least three cycles, suggesting high structural stability. This work presented the first example of azolate‐based MOFs showing a high NH_3_ uptake and stability after repeated measurements, which demonstrates the suitability of such MOFs as NH_3_ adsorbents.

**Figure 11:**
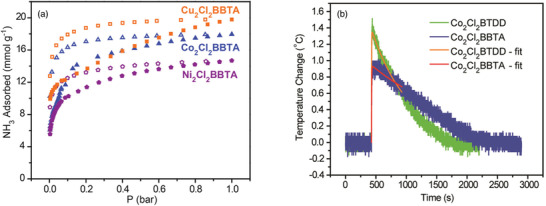
a) NH_3_ isotherms of activated Co_2_Cl_2_(BBTA) (blue triangles), Ni_2_Cl_2_(BBTA) (purple pentagons), and Cu_2_Cl_2_(BBTA) (orange squares) at 298 K. Closed and open symbols indicate adsorption and desorption, respectively. b) Infra‐SORP heat flux data of large‐pore Co_2_Cl_2_(BTDD) (green) and small‐pore Co_2_Cl_2_(BBTA) (blue) (NH_3_ 1000 ppm in N_2_ balance at a flow rate of 140 mL min^−1^). Reproduced with permission.^[^ ^82^ ^]^ Copyright 2018, American Chemical Society.

Following previous research, Rieth and Dincă additionally investigated the M_2_Cl_2_(BTTA) and M_2_Cl_2_(BTDD) (M = Mn, Co, Ni, and Cu) series for use as NH_3_ adsorbents.^[^ ^82^ ^]^ The M_2_Cl_2_(BTTA) frameworks have smaller pore sizes than those of M_2_Cl_2_(BTDD) owing to the shorter ligand length. In the NH_3_ isotherms at 298 K, the amounts of NH_3_ captured by Co_2_Cl_2_(BTTA), Ni_2_Cl_2_(BTTA), and Cu_2_Cl_2_(BTTA) were 17.95, 14.68, and 19.79 mmol g^−1^ at 1 bar, respectively (**Figure** **11**a). As compared with the M_2_Cl_2_(BTDD) MOFs which possesses a larger pore size, the M_2_Cl_2_(BTTA) MOFs exhibited higher NH_3_ uptakes. In particular, the adsorption capacity of Cu_2_Cl_2_(BBTA) was the highest among those reported for MOF‐based NH_3_ adsorbents. This result is attributed to the higher density of open metal sites and the cooperative proximity effects due to the smaller pores in isoreticular analogues. To analyze this phenomenon, a recent calorimetric technique, Infra‐SORP, was employed, and the relationship between the pore size of a framework and the NH_3_ sorption kinetics was investigated (Figure 11b). This work demonstrated that a desirable MOF‐based NH_3_ adsorbent can be prepared by controlling the pore size, which affects the NH_3_ uptake, kinetics, and structural stability of frameworks.

Janiak and co‐workers introduced a urea R‐NH‐CO‐CH‐R group by using 6‐oxo‐6,7‐dihydro‐5*H*dibenzo[*d*,*f*][1,3]diazepine‐3,9‐dicarboxylic acid (H_2_L1) as an organic linker in four zinc metal–organic frameworks and studied their gas adsorption capacity.^[^ ^83^ ^]^ The solvothermal reaction of H_2_L1 and the metal source Zn(NO_3_)_2_·6H_2_O in DMF yielded a twofold‐interpenetrated framework 3D‐[Zn_4_(*μ*_4_O)(L1)_3_] (**1**); the use of *N*,*N*‐diethylformamide (DEF) resulted in 2D‐[Zn_2_(L1)_2_(DEF)_2_·2.5DEF] (**2**). The pillaring of **2** was accomplished by replacing the DEF in the Zn paddle‐wheel cluster with a bridging ligand, 4,4′‐bipyridine (bipy) or 1,2‐bis(4‐pyridyl)ethane (bpe), yielding twofold interpenetrated structures of [Zn_2_(L1)_2_(bipy)] (**3**) and [Zn_2_(L1)_2_(bpe)] (**4**), respectively. Among the adsorbents, **3** and **4** showed high NH_3_ uptakes of 17.79 and 14.31 mmol g^−1^ at 273 K, respectively, with a large hysteresis originating from the multiple hydrogen bonds between the interpenetrated networks. This work used a novel and rigid urea‐functionalized organic linker in the synthesis of MOF to increase the number of hydrogen bonds for capturing harmful gases.

Structural integrity upon exposure to corrosive gases is of utmost importance for real applications. Yang and co‐workers studied a robust metal–organic framework, MFM‐300(Al), with exceptional reusability.^[^ ^84^ ^]^ The Al‐MOF, [Al_2_(OH)_2_(L)], was constructed with [AlO_4_(OH)_2_] components connected by 3,3′,5,5′‐biphenyl‐tetracarboxylic acid (H_4_L). The diameter of the channels in the MOF was ≈6.5 Å, and the hydroxyl groups were oriented toward the interior of the pores. The robustness of the MOF was tested with SO_2_ and NO_2_ in prior works.^[^ ^85^, ^86^, ^87^ ^]^ The NH_3_ isotherms were collected at 273 and 303 K, where the NH_3_ uptakes of MFM‐300(Al) were 15.7 and 13.9 mmol g^−1^, respectively. Remarkably, the NH_3_ capacity of the MOF was maintained over 50 adsorption–desorption cycles without a capacity loss. Additionally, the time to reach saturation in the adsorption cycle was ≈6 min and that of the desorption cycle was ≈13.5 min, implying that this MOF is an effective adsorbent for NH_3_ storage. The three binding sites of NH_3_ in the MOF were elucidated via in situ neutron powder diffraction and Rietveld refinement. The result was corroborated by in situ synchrotron FT‐IR microspectroscopy for the reversibility of H–D exchange, demonstrating the predominance of pseudo‐chemisorption binding. This study, which examined the NH_3_ binding sites in MOFs, spotlighted the strong potential of robust MFM‐300(Al) for NH_3_ storage.

Recently, Farha and co‐workers reported the NH_3_ adsorption capacity of a zirconium‐based MOF, NU‐300, which was uniquely connected with a low‐symmetry tricarboxylate organic linker.^[^ ^88^ ^]^ NU‐300 was constructed with the metal cluster Zr_6_O_8_ (four types of Zr^4+^ atoms) and distinctively 8‐coordinated with oxygen atoms from 3,5‐di(4′‐carboxylphenyl)benzoic acid (H_3_L), DMF, and formic acid. To study its chemical stability, NU‐300 was soaked in H_2_O at 100 °C, in 0.01 m aqueous HCl and in 0.001 m aqueous NaOH solutions for 24 h; the crystallinity of NU‐300 was retained under all conditions. In the consecutive cycles of NH_3_ adsorption and desorption at 298 K, the initial adsorption reached 8.28 mmol g^−1^ at 1.0 bar, and the uptake remained as high as 4 mmol g^−1^ at 0.1 bar, and it was 1.5 mmol g^−1^ at 0.01 bar. Moreover, the almost identical adsorption curves of successive cycles below 0.01 bar suggest the recyclability of NU‐300 at low pressures. The free carboxylate groups (**—**COOH) in NU‐300 provide acid–base interaction sites that strongly bind NH_3_ even at low pressures. The observation of the N–H vibration in NH_4_^+^ at 1480 cm^−1^, in in situ IR spectroscopy, upon adsorption and desorption verified the strong Brønsted acid–base reaction between the NH_3_ and carboxylate groups. This work presented the benefits of introducing uncoordinated Brønsted acid sites to MOFs for the capture of NH_3_ at low concentrations.

**Figure 12:**
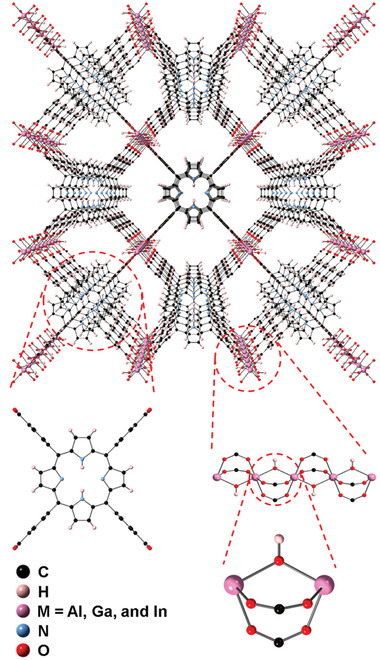
Crystal structure of M‐PMOF (M = Al, Ga, and In) with porphyrin linker, metal node, and Brønsted bridging OH group. Reproduced with permission.^[^ ^89^ ^]^ Copyright 2019, American Chemical Society.

Farha and co‐workers investigated the NH_3_ uptake capacity of isoreticular *meso*‐tetra(4‐carboxylphenyl)porphyrin (TCPP)‐based MOFs with bridging hydroxyl groups, that is, M_2_(OH)_2_(TCPP) (M = Al, Ga, and In), represented as Al‐PMOF, Ga‐PMOF, and In‐PMOF (**Figure** **12**).^[^ ^89^ ^]^ The metal nodes in the three MOFs form rod‐packing chains ([M(OH)(−COO)_2_]_∞_), which are connected by the porphyrin linker to create 1D channels with a width of ≈1 nm. From the NH_3_ adsorption isotherm obtained at 298 K for two consecutive cycles, it was observed that Al‐PMOF exhibited similar uptakes of 7.67 and 7.34 mmol g^−1^ in the first and second cycles, respectively. On the contrary, a reduced adsorption capacity was apparent for Ga‐PMOF and In‐PMOF with uptakes decreasing from 10.50 to 7.71 and 9.47 to 7.83 mmol g^−1^, respectively. Moreover, the steeper uptakes observed for Ga‐PMOF and In‐PMOF below 0.1 bar further suggest a stronger interaction with NH_3_ compared to that of Al‐PMOF. Indeed, diffuse reflectance infrared Fourier transform spectroscopy (DRIFTS) measurements in air at 294 K revealed the strongest O–H bonding in Al–OH–Al with the —OH stretch frequency at 3706 cm^−1^, followed by Ga–OH–Ga (2668 cm^−1^) and In–OH–In (3661 cm^−1^). In the difference spectra after NH_3_ exposure at 323 K, negative peaks were observed for Al‐PMOF, Ga‐PMOF, and In‐PMOF at 3700, 3659, and 3635 cm^−1^, respectively; these peaks correspond to the loss of —OH stretch vibrations. The positive peaks due to NH_4_^+^ cation stretching, observed at 1385, 1365, and 1350 cm^−1^, imply that the Brønsted acidic bridging hydroxyls were consumed to produce ammonium salt. This research identified that the binding strength of the Brønsted acidic sites for NH_3_ could be controlled based on the metal identity of MOFs.

**Figure 13:**
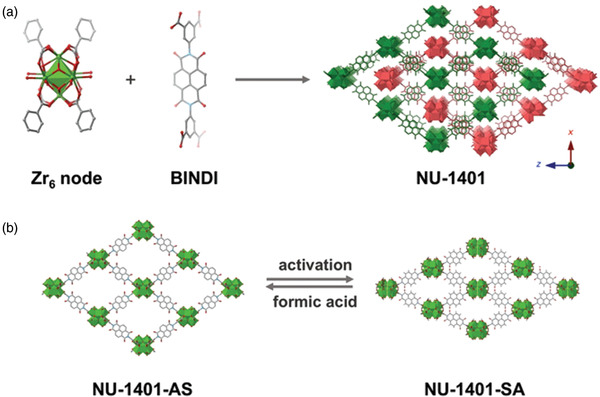
a) Crystal structure of NU‐1401 composed of Zr_6_ nodes and BINDI linkers. The different colors indicate interpenetrated nets. b) After activation by supercritical CO_2_, the single net structure in NU‐1401 changed (C: gray; O: red; Zr: green; H atoms were removed for clarity). Reproduced with permission.^[^ ^90^ ^]^ Copyright 2020, Wiley‐VCH.

The NH_3_ affinity of a flexible Zr‐MOF, NU‐1401, was studied by Farha and co‐workers.^[^ ^90^ ^]^ The MOF was constructed with 4‐connected Zr_6_ nodes and a 1,4,5,8‐naphthalenediimide‐based carboxylate organic linker, which comprises a twofold interpenetrated network (**Figure** **13**a). The activated structure by supercritical CO_2_ method is named NU‐1401‐SA. The BET surface area of NU‐1401‐SA was calculated to be 610 m^2^ g^−1^ with a total pore volume of 0.23 cm^3^ g^−1^ from Ar isotherms at 87 K (Figure 13b). The NH_3_ adsorption isotherm of NU‐141‐SA at 298 K displayed type‐I behavior, showing a sharp slope at low pressures. Furthermore, the amount of NH_3_ adsorbed was 8.41 mmol g^−1^ at 1 bar. From the breakthrough experiments at 2976 ppm under 80% RH, the NH_3_ capacity of NU‐1401 was estimated to be 5.7 mmol g^−1^. After the adsorption experiments, the changes in the diffraction peaks of the MOF were not observed in the PXRD patterns. This work suggests that the design and synthesis of interpenetrated Zr‐MOFs with flexibility could create new opportunities for air purification.

### 2.2 Other Porous Hybrid Adsorbents

**Figure 14:**
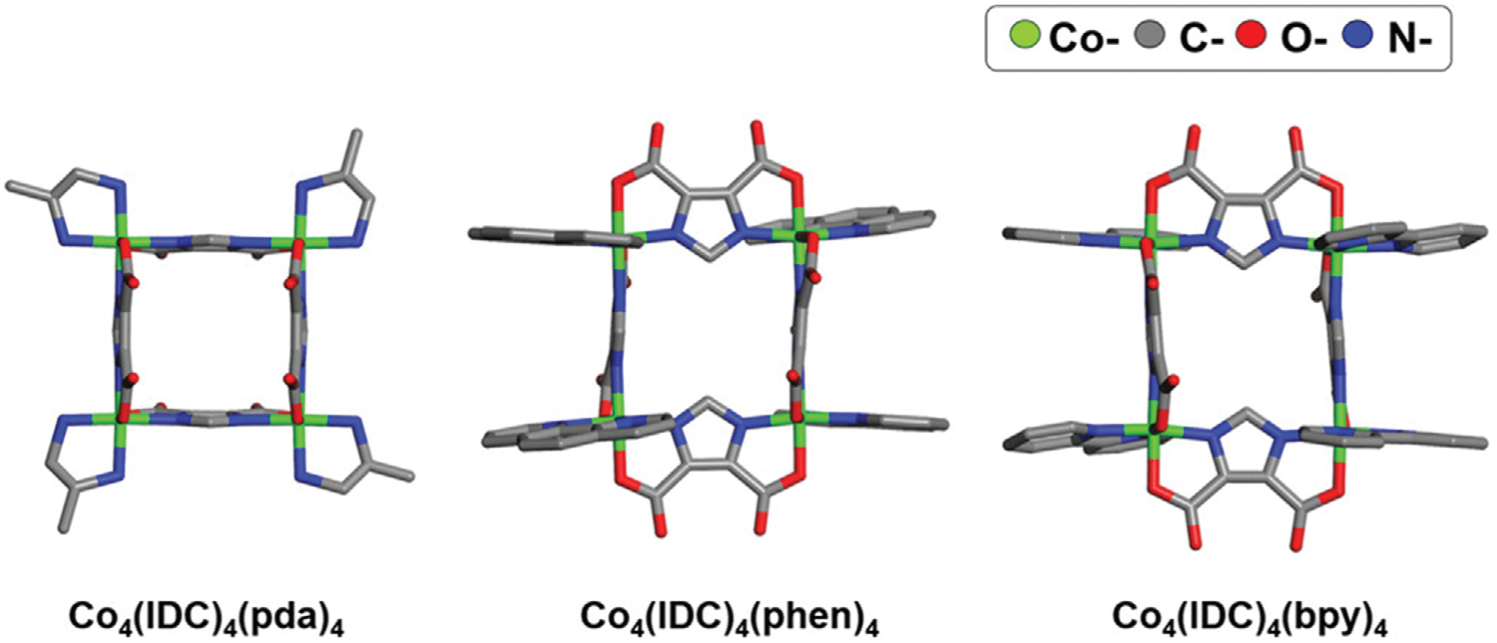
Crystal structure of metal–organic square adsorbing NH_3_. Reproduced with permission.^[^ ^91^ ^]^ Copyright 2017, American Chemical Society.

In addition to the MOFs investigated in the aforementioned studies, several inorganic–organic hybrid adsorbents, including metal–organic squares and pigments, have been explored for NH_3_ removal. Such materials exhibited unexpected performance that has not been observed in MOF adsorbents. Yang and co‐workers reported H_2_O/NH_3_ uptakes using reported metal–organic squares (MOSs).^[^ ^91^ ^]^ Co_4_(IDC)_4_(pda)_4_, (Co_4_(IDC)_4_(phen)_4_), and Co_4_(IDC)_4_(bpy)_4_ (IDC = 4,5‐imidazoledicarboxylate, pda = 1,2‐diaminopropane, phen = 1,10‐phenanthroline, bpy = 2,2′‐dipyridyl) were prepared and designated as MOS‐1, MOS‐2, and MOS‐3, respectively (**Figure** **14**).^[^ ^92^, ^93^ ^]^ This square‐like structure with supramolecular pores showed high structural stability because the metal cluster center of the MOS was protected by four dinitrogen ligands. Based on this property, the squares were expected to have advantages in the uptake of H_2_O/NH_3_ with respect to reusability. The BET surface areas of the MOSs were calculated to be 1112 (MOS‐1), 76 (MOS‐2), and 27 m^2^ g^−1^ (MOS‐3), respectively. The NH_3_ uptake of MOS‐1 was found to be 11.5 mmol g^−1^ at 25 °C and 1 bar. This adsorption is based on physisorption, depending only on the pressure, without hysteresis. MOS‐2 with a low BET surface area exhibited a low uptake of 5.2 mmol g^−1^ under the same conditions. In contrast, MOS‐3 showed a two‐step isotherm. In the first step, the NH_3_ capacity was low (<1 mmol g^−1^) below 0.3 bar; subsequently, the capacity increased to 3.8 mmol g^−1^. This phenomenon can be explained by the gate opening effect because the pore size of MOS‐3 (≈3 Å) is similar to the size of the kinetic diameter of NH_3_ (≈2.9 Å). Interestingly, the NH_3_ capacities of all the MOSs were consistent over 5 adsorption–desorption cycles. This work represents the first use case of metal–organic squares as potential NH_3_ adsorbents.

**Figure 15:**
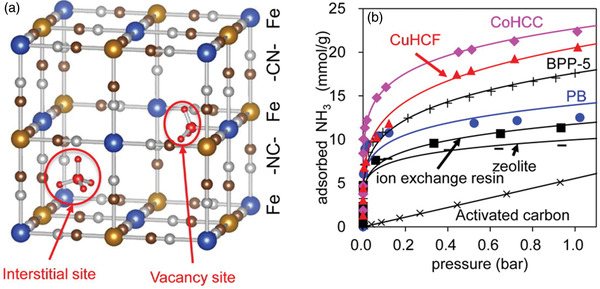
a) Crystal structure of Prussian blue (PB). b) NH_3_ isotherms of Prussian blue, copper hexacyanoferrate (CuHCF), cobalt hexacyanocobaltate (CoHCC), and other adsorbents at 298 K. Reproduced with permission.^[^ ^94^ ^]^ Copyright 2016, American Chemical Society.

Kawamoto and co‐workers focused on the adsorption abilities of Prussian blue (K_0.23_Fe[Fe(CN)_6_]_0.74_) and its analogues, Co(HCC) (Co[Co(CN)_6_]_0.60_) and Cu(HCF) (Cu[Fe(CN)_6_]_0.50_).^[^ ^94^ ^]^ These compounds have two types of NH_3_ adsorption sites (vacancies and interstitial sites), which can capture both NH_3_ and NH_4_^+^ (**Figure** **15**a). The NH_3_ adsorption isotherms of the samples were collected at 25 °C. The NH_3_ adsorption capacities of Prussian blue, Co(HCC), and Cu(HCF) were determined to 12.5, 21.9, and 20.6 mmol g^−1^, respectively, which are the highest values among porous and open adsorbents (Figure 15b). In particular, the adsorption rate of CoHCC is superior to that of ion‐exchange resins and zeolites. The XRD peaks of the samples remained unchanged before and after NH_3_ adsorption, indicating high framework stability. Notably, the excellent NH_3_ capture ability of Prussian blue, even in the condition of NH_3_ in ambient air (≈15 ppbv NH_3_), was confirmed via the time‐dependent IR spectra of Prussian blue, in which the peak (due to symmetric deformation of NH_4_^+^) at 1410 cm^−1^ had changed.

Following the previous work, Takahashi et al. studied Prussian blue (Na_0.05_Fe[Fe(CN)_6_]_0.70_·5.3H_2_O) and two other analogues, CuBPA (K_0.05_Cu[Fe(CN)_6_]_0.46_·5.0H_2_O) and CoPBA (K_0.05_Co[Co(CN)_6_]_0.66_·4.4H_2_O), for the removal of trace NH_3_ from the atmosphere.^[^ ^95^ ^]^ Co(PBA) was obtained from cobalt(II) chloride and potassium hexacyanocobaltate, and Cu(PBA) was prepared from copper sulfate and potassium ferrocyanide in a micromixer with a flow rate of 20 mL min^−1^ and 250 µm of hole meter.^[^ ^96^ ^]^ To evaluate the NH_3_ removal ability under trace levels of NH_3_ (10 ppmv), the breakthrough curves of the materials were recorded at 25 °C. The adsorption capacities of Prussian blue and CoBPA were 3.1 and 1.9 mmol g^−1^, respectively; for comparison, those of ion exchange resins, Zeolite 13X, and activated carbon were 0.38, 0.28, and 0.02 mmol g^−1^, respectively, at the same conditions. Interestingly, the adsorbents were easily regenerated by flowing 1 mL min^−1^ of H_2_O for 20 min; only by flushing water, 84% and 100% of the adsorbed NH_3_ were desorbed for Prussian blue and CoBPA, respectively. These works demonstrate that Prussian blue and its analogues have excellent NH_3_ uptake abilities even at trace NH_3_ levels, and that Prussian blue and its analogues are anticipated to become effective NH_3_ adsorbents with easy regeneration features, due to their interaction with H_2_O.

## 3 Porous Organic Materials

Few porous organic NH_3_ adsorbents connected by covalent or hydrogen bonds among the organic moieties have been developed. As COFs and POPs have a high structural stability owing to their covalent bonds, they exhibit high reusability. To increase the NH_3_ capture ability, various synthetic strategies, which cannot be employed in the case of metal–organic frameworks because of their structural collapse during the modification process, can be applied to COFs and POPs. For instance, direct postsynthetic acidification of POPs is an easy and useful method to obtain desirable NH_3_ adsorbents. Interestingly, HOFs displayed new characteristics, such as type‐IV isotherms for NH_3_ adsorption, which have not been observed in other porous materials.

### 3.1 Covalent Organic Frameworks

**Figure 16:**
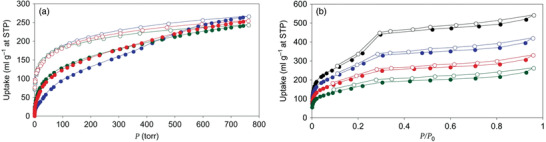
a) First (blue), second (red), and third (green) NH_3_ isotherms of COF‐10 at 298 K. b) N_2_ isotherms at 77 K after each NH_3_ isotherm measurement. All measurements were performed using the same COF‐10 adsorbent. Reproduced with permission.^[^ ^97^ ^]^ Copyright 2010, Springer Nature.

COFs are 2D or 3D porous crystalline solids where the organic building blocks are connected by covalent bonds. Although only a few studies that deal with the NH_3_ capture properties of COFs have been reported so far, COFs have potential in terms of structural tunability. The removal of NH_3_ using COF materials was first studied by Yaghi and co‐workers.^[^ ^97^ ^]^ They discovered that the NH_3_ adsorption capability of COF‐10 (15 mol kg^−1^ at 1 bar and 298 K) was higher compared to that of previously reported porous materials such as Zeolite 13X (9 mol kg^−1^), Amberlyst 15 (11 mol kg^−1^), and mesoporous silica MCM‐41 (7.9 mol kg^−1^). In COF‐10, hexahydroxytriphenylene (HHTP) and biphenyldiboronic acid (BPDA) form hexagonal layers that are stacked to construct 1D pores with a diameter of 34 Å. The Lewis acidic boron elements in the backbone provide strong interaction sites for NH_3_ adsorption. After two adsorption/desorption cycles, the NH_3_ adsorption capacity decreased only by 4.5% (**Figure** **16**). During the first cycle, the mesoporosity of COF‐10 was confirmed through type‐IV N_2_ adsorption isotherms. The microporosity increased after repeated NH_3_ sorption cycles. The authors explained that the NH_3_ adsorption in the interlamellar region of COF‐10 resulted in a stacking disorder upon removal; this is supported by the observation of hysteresis at low pressures during desorption. Thus, the variation in the low‐angle peaks of the PXRD pattern for COF‐10 indicates that complete structural disorder does not occur. The broadening and decreased intensities of the peaks are suggestive of increasing disorder in the packing between the layers. Binder‐free tablets of COF‐10 produced under 2000 psi showed an adsorption capacity comparable to that of the powder form. This research pioneered the application of covalent organic frameworks in NH_3_ capture.

**Figure 17:**
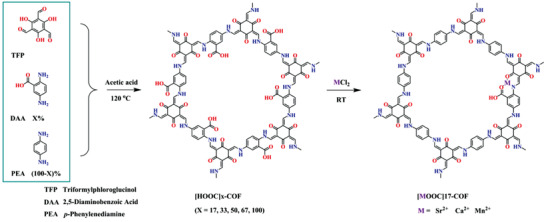
Scheme for the preparation of COFs with different compositions. Reproduced with permission.^[^ ^50^ ^]^ Copyright 2018, American Chemical Society.

Recently, [HOOC]*_x_*‐COFs (X = 0, 17. 33, 50, and 100) synthesized from COFs with varying ratios of triformylphloroglucinol (TFP), 2,5‐diaminobenzoic acid (DAA), and *p*‐phenylenediamine (PA‐1) were investigated by Yuan and co‐workers. (**Figure** **17**).^[^ ^50^ ^]^ Among the prepared COFs, [HOOC]_17_‐COFs exhibited the highest capacity of 9.34 mmol g^−1^ at 298 K and 1 bar. To further enhance the NH_3_ adsorption capacity of the best‐performing sample, the authors incorporated Ca^2+^, Mn^2+^, and Sr^2+^ on the pore surface to provide open metal sites with a strong Lewis acidity. The NH_3_ adsorption capacities of the metal–ion‐incorporated COFs were 12.25 ([CaOOC]_17_‐COF), 11.38 ([MnOOC]_17_‐COF), and 14.30 mmol g^−1^ ([SrOOC]_17_‐COF), under the same conditions. The XPS (X‐ray photoelectron spectroscopy) results of [HOOC]_0_‐COF, [HOOC]_17_‐COF, and [SrOOC]_17_‐COF exposed to NH_3_ showed reduced binding energies of N 1s and O 1s compared to those of the activated COF samples, indicating the formation of hydrogen bonds with the nitrogen and oxygen atoms in the frameworks after NH_3_ adsorption. Moreover, the XPS peaks arising from the ammonium salt at 401.0 eV for [HOOC]_17_‐COF and 400.8 eV for [SrOOC]_17_‐COF suggested that acid–base reactions occurred between NH_3_ and carboxylic acid. The decrease in the binding energy (133.7 eV) of Sr in NH_3_‐adsorbed samples from 134.5 eV for SrCl_2_ and from 134.2 eV for the activated samples verified the coordination of NH_3_ to Sr. The binding affinity of the acidic functional groups for NH_3_ increased in the order of —NH, —C=O, —COOH, and metal ions, as supported by IR measurements at different temperatures where desorption occurred, that is, at 303, 323, 363, and 423 K, respectively. This research suggests that the NH_3_ adsorption capacity can be increased by introducing various metal binding sites through the surface modification of COFs.

### 3.2 Porous Organic Polymers

**Figure 18:**
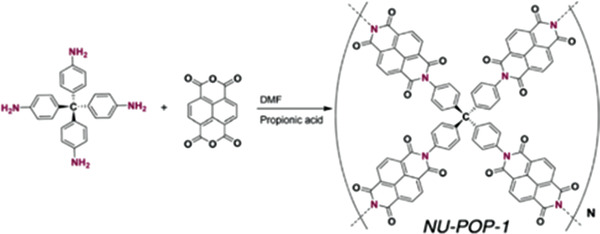
Synthesis scheme for NU‐POP‐1. Reproduced with permission.^[^ ^98^ ^]^ Copyright 2012, Springer Nature.

The high structural robustness of amorphous POPs, associated with strong covalent bonds between the organic moieties, is a positive attribute for the removal of NH_3_. Owing to this stability, many acidic functional groups can be densely introduced into the backbone of POPs, resulting in high adsorption of NH_3_. Hupp and co‐workers investigated the adsorption capability of NU‐POP‐1 (NU = Northwestern University) on NH_3_, cyanogen chloride, sulfur dioxide, and octane, as shown in **Figure** **18**.^[^ ^98^ ^]^ The adsorption properties were measured through microbreakthrough tests under dry and 80% RH conditions. Under the same conditions, the adsorption capacities were compared with those of BPL (Calgon Carbon Corporation; Zn/BPL/TEDA) that was doped with ZnO and triethylenediamine to enhance the hydrolytic decomposition of cyanogen chloride. To examine the hydrophilicity of the structures, ambient‐temperature H_2_O adsorption isotherms were recorded for NU‐POP‐1 and Zn/BPL/TEDA. Higher H_2_O uptake was observed for NU‐POP‐1 below 60% RH due to the smaller pore sizes and the presence of O and N sites within the pores. On the other hand, the greater pore volume of carbon (0.5 cc g^−1^) compared to that of NU‐POP‐1 (0.32 cc g^−1^) led to a greater H_2_O loading at increased humidity. In the NH_3_ breakthrough measurement of NU‐POP‐1, the saturation loading was 5.56 mol kg^−1^ under dry conditions and 6.17 mol kg^−1^ under humid conditions, indicating that NH_3_ can penetrate into the pores even with the presence of H_2_O. In comparison, Zn/BPL/TEDA displayed saturation loadings of 0.69 and 0.45 mol kg^−1^ in dry and humid conditions, respectively. The superior capacity of NU‐POP‐1 could be attributed to the nitrogen and oxygen groups that provide adsorption sites for NH_3_ or tight pores (diameters of 3.5, 5.2, and 8.2 Å) as discovered by pore size analysis. In both materials, NH_3_ was completely removed after the feed terminated, suggesting the physical adsorption of NH_3_. This study revealed that POP without reactive moieties could capture toxic chemicals in both dry and humid conditions more efficiently than impregnated activated carbon.

Long and co‐workers reported the synthesis and NH_3_ adsorption features of diamondoid porous organic polymers with isolated and cooperative Brønsted acidic substituents.^[^ ^99^ ^]^ The parent framework PAF‐1 was synthesized through Yamamoto polymerization, nitrated under the Menke condition, and then reduced with sodium dithionite to produce [(C_6_H_4_‐C_6_H_3_NH_2_)_2_(C)] (BPP‐1: Berkeley porous polymer‐1). Addition of HCl to BPP‐1 afforded [(C_6_H_4_‐C_6_H_3_NH_3_Cl)_2_(C)] (BPP‐2). PPN‐6‐SO_3_H with sulfonic acid groups attached to the backbone of PAF‐1 was prepared after PAF‐1 was treated with chlorosulfonic acid. The NH_3_ adsorption capacity of PAF‐1 and its derivatives at 298 K unambiguously improved with the increasing acidity of the functional groups (PPN‐6‐SO_3_H > BPP‐2 > BPP‐1 > PAF‐1) at both the low pressure of 500 ppm and high pressure of 1 bar regardless of the calculated BET surface area. The same polymerization procedure performed on tetrakis(3‐formyl‐4‐bromophenyl)silane yielded [(2,2′‐C_6_H_3_CHOC_6_H_3_CHO)_2_(Si)] (BPP‐3), the interpenetrated structure of which was indicated by the low N_2_ adsorption at 77 K and the smaller pore size than that of the open pore structures. The authors explained that dipole–dipole interactions between the functional groups lead to attractive forces between separate networks, which resulted in interpenetrated structures with controlled spatial sizes. In the cases of [(C_6_H_4_‐*p*‐C_6_H_2_(CO_2_CH_3_)_2_‐C_6_H_4_)_2_(C)] (BBP‐4) and [(C_6_H_4_‐*p*‐C_6_H_2_(CO_2_ *n*‐C_9_H_19_)_2_‐C_6_H_4_)_2_(C)] (BPP‐6), their monomers were synthesized through the esterification of dibromoterephthalic acid with methanol for BPP‐4 or 1‐nonanol for BPP‐6 followed by Miyaura borylation. Then, the monomers and terakis(4‐bromophenyl)methane were polymerized through the Suzuki reaction with Buchwald's palladacycle precatalyst to prepare BPP‐4 and BPP‐6, respectively. Additionally, [(C_6_H_4_‐*p*‐C_6_H_2_(CO_2_H)_2_C_6_H_4_)_2_(C)] (BPP‐5) was obtained after the saponification of BPP‐4 under basic conditions followed by reacidification. Similarly, [(C_6_H_4_‐*p*‐C_6_H_2_(CO_2_H)_2_C_6_H_4_)_2_(C)] (BPP‐7) was prepared after the side chain cleavage from BPP‐6. Together with small pore sizes of 5.4–5.6 Å for BBP‐5 and 6.0–6.5 Å for BPP‐7, the absence of bromine via energy dispersive X‐ray (EDX) spectroscopy verified the interpenetrated structures. The superior NH_3_ uptake of the less acidic carboxylic acid functionalized polymers compared to that of PPN‐6‐SO_3_H in both low and high pressure proved that the cooperative interaction in the interpenetrated structure enhanced the NH_3_ adsorption capacity. Moreover, the NH_3_ adsorption (17.7 mmol g^−1^) of BPP‐5 exceeded that (16.1 mmol g^−1^) of BPP‐7 at 1000 mbar. Overall, the results indicate that the spatial arrangement of the acidic sites in porous organic networks allows for cooperative behavior, resulting in enhanced NH_3_ adsorption.

**Figure 19:**
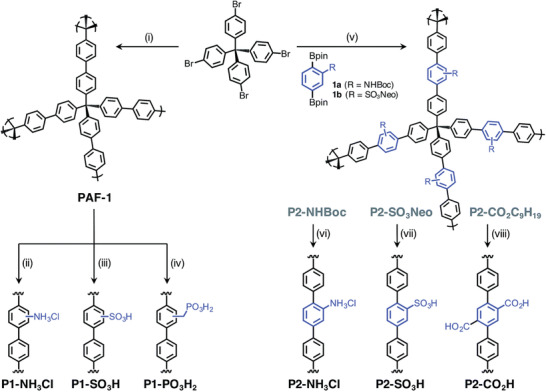
Synthesis scheme for P1 and P2 porous organic polymers with different Brønsted acid groups. Reproduced with permission.^[^ ^49^ ^]^ Copyright 2017, The Royal Society of Chemistry.

Following the previous study, Long and co‐workers reported six Bronsted acidic POPs containing —NH_3_Cl, —CO_2_H, —SO_3_H, and PO_3_H_2_ groups on the non‐interpenetrated (P1) and interpenetrated (P2) frameworks (**Figure** **19**).^[^ ^49^ ^]^ The P1 series was prepared by postsynthetic modifications of the high‐surface area porous aromatic polymer PAF‐1, and the P2 series was synthesized through Suzuki coupling polymerizations of acidic monomers via protection–deprotection processes. When the NH_3_ adsorption capacity was examined up to 1 bar at 298 K under dry conditions, the polymers functionalized with —NH_3_Cl showed a generally low adsorption capacity as expected from the lower Bronsted acidity. However, the NH_3_ uptake at a low pressure of 0.05 mbar (50 ppm) was not directly correlated with acidity, and the values for P1‐SO_3_H, P1‐PO_3_H_2_, P2‐SO_3_H, and P2‐CO_2_H were 0.01, 2.03, 1.79, and 1.62 mmol g^−1^, respectively. Although sulfonic acid has lower pKa values than phosphonic acid, the number of acidic protons, smaller surface area and pore volume, bulkiness, and flexibility of the acid functional group in P1‐PO_3_H_2_ provide more available and proximal acid sites for NH_3_ adsorption. Moreover, the interpenetrated network of P2 polymers enables local dielectric polarization in the acidic pores, leading to enhanced interaction with NH_3_. The adsorption results of P1‐PO_3_H_2_ and P2‐CO_2_H suggest that the effect of the high density of weak acidic sites in a confined space is comparable to that of the presence of strong acidic sites, with regard to NH_3_ capture. Adsorption capacities measured via using dynamic breakthrough measurements under dry conditions were in accordance with those obtained by NH_3_ isotherms (saturated NH_3_ loadings were as high as 5.2 and 6.7 mmol g^−1^ for P1‐PO_3_H_2_ and P2‐CO_2_H, respectively). Under an 80% RH condition, the saturation capacity of all samples increased, and outstanding adsorption capacities of 8.1, 7.2, and 7.4 mmol g^−1^ were achieved for P1‐SO_3_H, P1‐PO_3_H_2_, and P_2_‐CO_2_H, respectively. In situ FT‐IR verified the proton transfer reaction between NH_3_ and acid sites, showing distinct ammonium ion peaks and deprotonated acid peaks upon NH_3_ adsorption. This study demonstrated the influence of structural and chemical properties on NH_3_ capture at low pressures in porous organic polymers, which enables rational designs of NH_3_ adsorbents.

**Figure 20:**
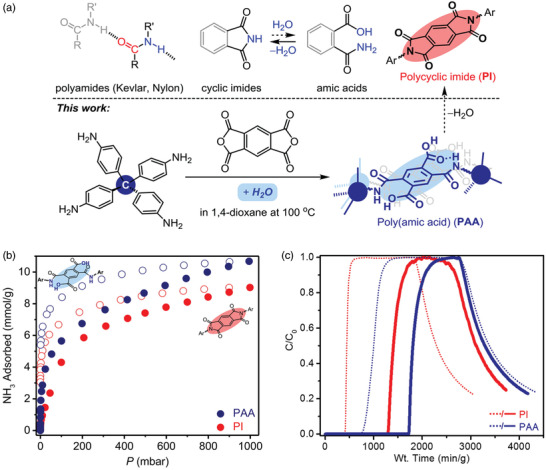
a) Scheme for the synthesis of PAA and PI. PI was obtained in the absence of water. b) NH_3_ isotherms of PI (red) and PAA (blue) at 298 K. c) NH_3_ breakthrough curves of PAA and PI in a stream of 2000 mg m^−3^ NH_3_ under dry (dashed lines) and 80% RH (solid lines) at 298 K. Reproduced with permission.^[^ ^100^ ^]^ Copyright 2017, American Chemical Society.

The same group also reported the one‐step, catalyst‐free synthesis of porous poly(amic acid) (PAA), which captured NH_3_ efficiently with Brønsted acidic sites (—COOH), proton donors (—CONH—), and proton acceptors (C=O), as shown in **Figure** **20**.^[^ ^100^ ^]^ PAA was prepared by dissolving tetrakis(4‐aminophenyl)methane and pyromellitic anhydride in H_2_O/1,4‐dioxane mixture (5% H_2_O, v/v) at 100 °C for 18 h. Here, the addition of H_2_O in the solvent is crucial to prevent the formation of porous polycyclic imide (PI) via dehydration. The NH_3_ adsorption isotherm of PAA at 298 K showed steeper and higher NH_3_ uptake (10.7 mmol g^−1^ at 1 bar) than that of PI (9.0 mmol g^−1^ at 1 bar) despite the lower BET surface area and smaller pore volume of PAA (365 m^2^ g^−1^) than those of PI (725 m^2^ g^−1^). In particular, PAA exhibited an NH_3_ uptake (1.6 mmol g^−1^) four times greater than that of PI (0.4 mmol g^−1^) at 1 mbar. Notably, NH_3_ capture was characterized through dynamic microbreakthrough measurements at 298 K, demonstrating a higher adsorption capacity of 4.4 mmol g^−1^ for PAA and 3.4 mmol g^−1^ for PI in humid (80% RH) conditions compared to 2.4 mmol g^−1^ for PAA and 1.1 mmol g^−1^ for PAA in dry conditions. Under humid conditions, H_2_O molecules assisted the formation of additional hydrogen bonding networks, resulting in enhanced saturation capacities of both PI and PAA because H_2_O facilitates proton transfer from carboxylic acids to NH_3_ (in PAA) or the dissolution of NH_3_ (in PI). This study is the first reported investigation of the porous poly(amic acid) polymer and its application to NH_3_ capture. Particularly, the role of H_2_O molecules in NH_3_ capture by POPs was intensively investigated.

### 3.3 Hydrogen‐Bonded Organic Frameworks

**Figure 21:**
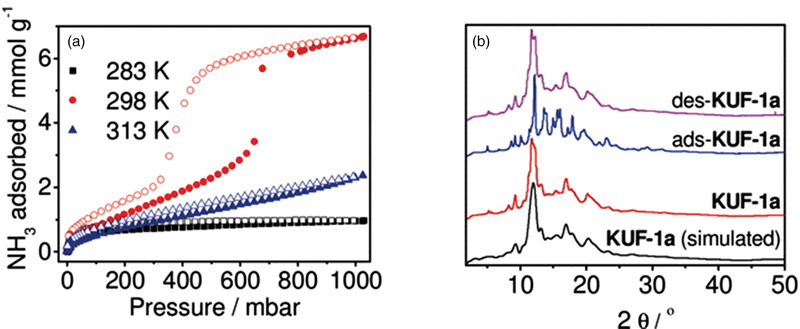
a) NH_3_ isotherms of KUF‐1a at 283, 298, and 313 K. Filled and nonfilled symbols denote adsorption and desorption, respectively. b) PXRD patterns of simulated KUF‐1a, KUF‐1a, and NH_3_‐adsorbed KUF‐1a (ads‐KUF‐1a) and NH_3_‐desorbed KUF‐1a (des‐KUF‐1a). Reproduced with permission.^[^ ^103^ ^]^ Copyright 2019, Wiley‐VCH.

Recently, HOFs based on the construction of hydrogen bonding networks have been extensively applied in gas separation, catalysis, and proton conductivity.^[^ ^40^, ^101^, ^102^ ^]^ In 2019, Hong and co‐workers introduced a new HOF known as the Korea University Framework‐1, KUF‐1, as a potential NH_3_ adsorbent that exhibits an S‐shaped (type IV) NH_3_ adsorption pattern.^[^ ^103^ ^]^ KUF‐1 retains its structure via hydrogen bonds between the H atoms of the guanidium cations (GuaH^+^) and the O atoms in the sulfonate of SPM^4−^ (H_4_SPM: tetrakis(4‐sulfophenyl)methane) in the orthorhombic crystal system (C222_1_). After the degassing process, the structure of KUF‐1 changed to that of KUF‐1a with the monoclinic system (P2_1_), whose structure was elucidated via the Rietveld refinement. Although no apparent gas adsorption was observed in the isotherms of N_2_, H_2_, and O_2_ due to nonporosity, the NH_3_ isotherms of KUF‐1a showed a distinct S‐shaped curve at 298 K, with amount adsorbed rising from 6.67 mmol g^−1^ at 1 bar (0.97 mmol g^−1^ at 283 K) (**Figure** **21**). The PXRD patterns revealed that the structure was altered and reconstructed after NH_3_ adsorption. A reversible structural change occurred during adsorption–desorption. Notably, regeneration was achieved in vacuum at room temperature, unlike in previous works, which commonly required harsh conditions. The adsorption capacity was retained after five cycles. This research is the first reported investigation of NH_3_ adsorption using a HOF for which a type‐IV isotherm was observed to accompany the rearrangement of the framework during the NH_3_ sorption process.

Li and co‐workers reported pyrene‐based HOFs (HOF‐100, HOF‐101, and HOF‐102) with mesoporosity.^[^ ^104^ ^]^ HOF‐100, HOF‐101, and HOF‐101 were formed by 1,3,6,8‐tetracarboxy pyrene, 1,3,6,8‐tetrakis (*p*‐benzoic acid), and 1,3,6,8‐tetra(6‐carboxynaphthalene‐2‐yl) pyrene, respectively, through myriad hydrogen bonds between the carboxylic dimers and layer stacking by strong *π*–*π* interactions. In the case of HOF‐101, its crystal structure was evaluated using the single‐crystal X‐ray diffraction method, and the other structures were determined via molecular modeling. Based on this structural information, their pore sizes were estimated to be 0.8 × 1.2 nm^2^ (HOF‐100), 1.8 × 2.4 nm^2^ (HOF‐101), and 2.5 × 3.0 nm^2^ (HOF‐102). The pore size of HOF‐102 with a type‐IV isotherm was determined to be 2.8 nm from the Ar isotherm at 87 K, using a nonlocal density functional theory (NLDFT) model. Owing to the strong pi‐pi interaction, the crystallinity of HOF‐102 was maintained under boiling H_2_O, NaOH solutions (pH = 14), and 18 m H_2_SO_4_ solutions. The framework adsorbed 250 cm^3^ g^−1^ of NH_3_ gas at 25 °C and 1 atm with no structural collapse.

### 3.4 Other Porous Organic Adsorbents

Activated carbon has been widely utilized in industrial fields because of its simple synthetic procedures and economic benefits, which are important factors for mass production. Thus, the characteristics of adsorption of NH_3_ by various activated carbons and their modified materials have been studied. In the study carried out by Huang et al., HNO_3_, H_2_SO_4_, HCl, H_3_PO_4_, and CH_3_COOH at various concentrations (1, 6, and 12 n) were reacted with coconut shell‐based activated carbon to incorporate acidic functional groups on the surface of the carbons to investigate the relationship between the acidic oxides of activated carbon and NH_3_ uptake.^[^ ^105^ ^]^ The products were denoted as N‐AC (nitric acid), S‐AC (sulfuric acid), P‐AC (phosphoric acid), A‐AC (acetic acid), and C‐AC (hydrochloric acid). The incorporation of acidic functional groups was confirmed by IR data, and the total amounts of the acidic groups in the activated carbon were evaluated via the Boehm titration method. The amount of acidic groups in 12 n N‐AC was 2.064 mmol g^−1^, which is the highest value among the carbons investigated in this work. Furthermore, from the N_2_ isotherm data, the specific surface area (1123 m^2^ g^−1^) of 12N C‐AC, which is the highest value among the acid‐treated carbons, was found to be superior to that (1073 m^2^ g^−1^) of untreated activated carbon. To evaluate the NH_3_ uptake ability of the acidified carbons, NH_3_ breakthrough measurements were conducted under 10 000 ppm at room temperature. In proportion to the amount of acidic groups, 12 n N‐AC exhibited the highest uptake value (41.648 mg NH_3_ g^−1^ AC) when compared with that of other acid‐treated carbons.

Similarly, Foley and co‐workers reported functionalized nanoporous carbons for NH_3_ capture.^[^ ^106^ ^]^ Among the several carbons, NPC‐PEG‐AC was obtained via prolysis at 800 °C from NPC‐PEG, which was synthesized from polyethylene glycol (PEG) and polyfurfuryl alcohol (PFA). After nitric acid treatment, NPC‐PEG‐AC was modified to NPC‐PEG‐AC‐F with dense carboxylic acid groups. The amount of NH_3_ adsorbed by NPC‐PEG‐AC reached 10.2 mmol g^−1^ at 25 °C and 1 bar. Interestingly, the functionalized carbon showed an uptake (17.0 mmol g^−1^ at 25 °C and 1 bar) higher than that of the nonfunctionalized carbon. The high heat of NH_3_ adsorption (≈165 kJ mol^−1^) of NPC‐PEG‐AC‐F originated from the high density of carboxylic acid groups, which explains the enhanced NH_3_ uptake. These studies suggest that the NH_3_ capture ability of the activated carbon was easily enhanced by postsynthetic modifications such as strong acid treatment.

The Fe_3_C‐derived carbons for NH_3_ adsorption were prepared by Mangarella and Walton.^[^ ^107^ ^]^ After commercial Fe_3_C was carbonized from 200 to 1000 °C with chlorine gas, the resultant solid was annealed with H_2_ to produce Fe_3_C‐CDC‐CT‐H_2_, where CT and H_2_ indicate the chlorination temperature and exposure to H_2_, respectively. Based on the N_2_ isotherm data collected at 77 K, a relationship between the specific surface area of carbon and chlorination temperature was elucidated. The NH_3_ uptake ability of unannealed Fe_3_C‐CDCs was investigated through NH_3_ breakthrough measurements (dry condition: NH_3_ 1500 ppm; humid condition: NH_3_ 7155 ppm under 75% RH). As a result, all unannealed carbons exhibited similar breakthrough times of ≈1300 min g^−1^, except for Fe_3_C‐CDC‐600‐5h, which has the lowest Fe content. The NH_3_ working capacity of Fe_3_C‐CDC‐600‐0.5h with 8.4 wt% Fe was the highest under both dry (1.88 mmol g^−1^) and humid (3.44 mmol g^−1^) conditions when compared with that of Fe_3_C‐derived carbons. This work demonstrates that the FeCl_3_ nanoparticles play an important role in dynamic NH_3_ capture.

## 4 Porous Composite Materials

The fabrication of composites using MOFs is an effective strategy that focuses on overcoming shortcomings such as the low stability of MOFs and low NH_3_ uptake of other materials while exploiting the high NH_3_ uptake of MOFs and the high stability of other materials. Composite materials, therefore, have enhanced NH_3_ uptake, high NH_3_ selectivity, increased structural stability under humid conditions while retaining the original merits of porous materials. Although MOF composites have been reported to increase the structural stability of MOFs with a high NH_3_ adsorption capacity under humid conditions, porous organic material composites have been studied in recent years to obtain optimized material properties for NH_3_ capture applications.

**Figure 22:**
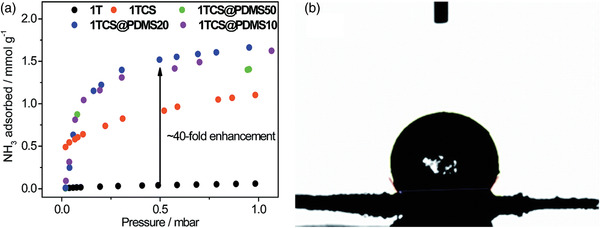
a) NH_3_ isotherms of PDMS‐coated samples at low pressures and 298 K. b) Water droplet test of 1TCS@PDMS10. Reproduced with permission.^[^ ^108^ ^]^ Copyright 2018, The Royal Society of Chemistry.

Hong and co‐workers enhanced the NH_3_ uptake of hypercrosslinked porous organic polymers (HCP) through double postsynthetic acidification and coating with hydroxyl‐terminated poly(dimethylsiloxane) (PDMS).^[^ ^108^ ^]^ The HCP (1T) was prepared by the solvothermal reaction of toluene, formaldehyde dimethyl acetal (FDA), 1,2‐dichloroethane (DCE), and the FeCl_3_ catalyst. The reaction time was shortened from 18–24 h to 5 h with a microwave‐assisted reaction.^[^ ^109^, ^110^ ^]^ The product 1T was further oxidized with KMnO_4_ and NaOH to yield carboxylic acid functionalized 1TC, which was then exposed to chlorosulfonic acid to yield sulfonated 1TCS. The NH_3_ adsorption capacity of 1T, 1TC, and 1TCS at 298 K and 1 bar was 3.8, 6.41, and 8.52 mmol g^−1^, respectively. In spite of their decreased BET surface areas, the gradual increase in the capacity is due to the increased amount of acidic functional groups in the materials. The high density of acidic sites played a significant role in the adsorption capacities at a low pressure of 500 ppm. To study the binding affinity of the pore surfaces of adsorbents with densities of acidic sites, the adsorbed amount of NH_3_ was divided by the surface area. Before the modifications, the NH_3_ adsorption capacity per surface area of 1T and 1TC was 4.372 × 10^−5^ and 6.159 × 10^−4^ mmol m^−2^, respectively, whereas that of 1TCS was 1.278 × 10^−2^ mmol m^−2^ at 0.5 mbar and 298 K, indicating the incorporation of high densities of acidic sites through double postsynthetic modifications. To enhance NH_3_ selectivity over H_2_O, the surface of 1TCS was coated with various amounts of hydroxyl‐terminated PDMS (**Figure** **22**). Remarkably, the coated PDMS imparted a hydrophobic character on the surfaces of 1TCS and provided more adsorption sites via the hydroxyl groups of PDMS. As a result, the adsorption of PDMS‐coated 1TCS (1.41 mmol g^−1^) was 40 times greater than that of 1T (0.04 mmol g^−1^) at 500 ppm. This work represents the first example of the use of double postsynthetic modification and hydrophobic polymer coating for NH_3_ capture using porous materials.

LeVan and co‐workers impregnated Cu‐BTC into MCM‐41, which is a well‐known ordered mesoporous silicate with a robust framework under high temperatures and humidities.^[^ ^111^ ^]^ After MCM‐41 was prepared, copper nitrate was impregnated into its pores (≈37 Å) via stirring in an aqueous solution. Then, 1,3,5‐tricarboxylic acid was reacted with Cu‐impregnated MCM‐41 to produce Cu‐BTC in the pores. The growth of the MOF in MCM‐41 was confirmed by scanning electron microscope (SEM) images and powder XRD patterns. From the N_2_ adsorption, the surface areas of Cu‐BTC, MCM‐41, and Cu‐MCM‐BTC were determined to be 1004, 809, and 836 m^2^ g^−1^, respectively. According to the breakthrough measurements at 1500 ppm under dry conditions, the NH_3_ adsorption capacities of Cu‐BTC, MCM‐41, and Cu‐MCM‐BTC were 9.0, 2.0, and 5.2 mmol g^−1^, respectively. These results demonstrate the positive effect of impregnated MOFs with regard to NH_3_ capture. Furthermore, under saturated water vapor, the adsorbed amounts changed to 1.5 (Cu‐BTC), 3.4 (MCM‐41), and 4.3 mmol g^−1^ (Cu‐MCM‐BTC). The degree of decomposition in the composite was lower than that of Cu‐BTC because of the presence of the silica matrix. This work indicates that composites comprising a MOF and mesoporous silica exhibit enhanced NH_3_ uptake under humid conditions.

Hydrophobic mixed‐matrix membranes (MMMs), including HKUST‐1 and polyvinylidene difluoride (PVDF), were studied by DeCoste et al. for NH_3_ removal.^[^ ^112^ ^]^ The membranes were denoted as [MOF wt%]‐HKUST‐1‐MMM, where wt% was 30, 40, or 67. For MMMs, increasing the content of MOF enhanced the intensities of the MOF peaks in the PXRD patterns. Before and after the NH_3_ exposure of HKUST‐1 powder, appreciable changes in the MOF phase were observed in the PXRD pattern and IR spectra, indicating the degradation of the framework. When the materials were exposed to 90% RH and 25 °C, the amount of NH_3_ adsorbed by the MOF declined considerably to below 10% of the initial capacity within a week while the NH_3_ capacities of 50‐HKUST‐1 and 67‐HKUST‐1 MMMs decreased by less than 20% even after 4 weeks. Although the intensities of the PXRD peaks were diminished, with unchanged peak positions, a new solid phase was only observed in the XRD pattern of the powder sample. These results indicate that the stability of a MOF in humid conditions was significantly enhanced via its mixing with a hydrophobic polymer.

Three MOF‐activated carbon composites (STAM‐17‐OEt@BPL_1, _2, _3 with MOF loadings of 77%, 51%, and 39%, respectively) were fabricated by McHugh et al.^[^ ^113^ ^]^ The exceptional hydrolytic stability of STAM‐17‐OEt, which has sacrificial bonds in the coordination of its metal centers, was reported by the same group.^[^ ^114^ ^]^ The MOF crystals were cultivated inside BPL activated carbon in situ. After 5‐ethoxy isophthalic acid and BPL activated carbon were dispersed in deionized water, copper acetate monohydrate was added to the mixture and refluxed for 3 d to produce STAM‐17‐OEt@BPL. With increasing contents of carbon, the PXRD peaks of the composites broadened due to the amorphous character of the carbon. The grown MOFs within the carbon were confirmed by SEM images. Using microbreakthrough measurements at 450 ppm, the NH_3_ adsorption capacity of the composites was investigated. Although the NH_3_ uptake of pure BPL carbon was only 0.43 wt%, that of STAM‐17‐OEt was 4.33 wt%, based on the weight of the material. Among the composites, STAM‐17‐OEt@BPL_1, which had the highest MOF loading, exhibited an NH_3_ adsorption of 1.78 wt%, suggesting that the NH_3_ amount adsorbed is proportional to the amount of loaded MOF. This work unveiled that the MOF‐carbon composites can purify contaminated air, including NH_3_ abatement.

**Figure 23:**
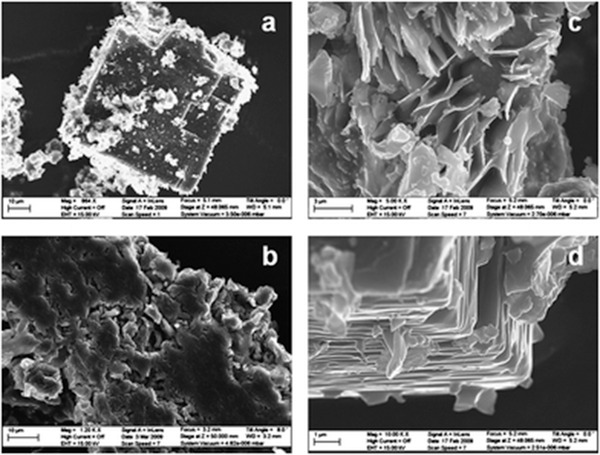
SEM images of a) MOF‐5, b) GO, and c,d) MOF‐5–GO composites. Reproduced with permission.^[^ ^117^ ^]^ Copyright 2009, The Royal Society of Chemistry.

In 2008, Petit and Bandosz reported that aluminum–zirconium oxy‐cations were impregnated into micro and mesoporous wood‐based activated carbon and calcinated at 300 °C.^[^ ^115^ ^]^ Based on NH_3_ breakthrough experiments, the aluminum–zirconium polycations were demonstrated to improve NH_3_ adsorption by supplying new Brønsted acidic sites that interact strongly with the adsorbed NH_3_ in its protonated form. In the next year, graphite oxide (GO) composites with polyoxometalate and MOF‐5 were reported.^[^ ^116^, ^117^ ^]^ Two Keggin polyanions, such as H_3_PW_12_O_40_ and H_3_PMo_12_O_40_, with high acidity were impregnated into GO with poly(diallydimethylammonium chloride). The impregnated polyoxometalate or functional groups of GO formed NH_4_^+^ with NH_3_, enabling enhanced NH_3_ uptake. Moreover, the composite MOF‐5–GO, comprising MOF‐5 and GO, was obtained by adding 5 wt% of GO during the synthesis of MOF‐5.^[^ ^117^ ^]^ The peaks of MOF‐5 and GO were observed in the PXRD pattern of the composite. Particularly, in the SEM images, the layers of MOF‐5 crystallites seemed to be separated from the layers of GO (**Figure** **23**). Through NH_3_ breakthrough measurements at 1000 ppm, it was found that the NH_3_ adsorption capacity of MOF‐5–GO was 6.9 and 53.5 mg g^−1^ under dry and 70% RH conditions, respectively, which is lower than that of pure GO with a high NH_3_ adsorption capacity (55.5 and 61.0 mg g^−1^). The high NH_3_ uptake of GO was explained via the acidic functional groups of GO and the intercalation of NH_3_ between distorted layers of GO. However, the adsorption capacity of the composite in the presence of moisture was 12% greater than the expected performance, which is owing to the structural synergy effect. In humid environments, H_2_O attacks and destroys the framework of MOF‐5; thus, NH_3_ can interact with the carboxylic groups of the organic linkers in MOF‐5, resulting in the synergy effect in the composite.

Following the study of MOF‐5–GO, the ratio of GO in the composite was modified to 5%, 10%, 20%, and 55%, to prepare MOF‐5/GO1, MOF‐5/GO2, MOF‐5/GO3, and MOF‐5/GO4, respectively.^[^ ^118^ ^]^ As the GO content increased, the NH_3_ breakthrough capacity varied from 7 mg g^−1^ (MOF‐5/GO1) to 82 mg g^−1^ (MOF‐5/GO4) under 1000 ppm of dried NH_3_; this capacity is higher than that (33 mg g^−1^) obtained via hypothetical calculations under the same conditions. This result was explained by three factors: i) intercalation between the layers of GO, ii) interfacial adsorption sites between MOF‐5 and GO (synergy effect), where enhanced dispersive forces are applied, and iii) hydrogen bonds with zinc oxide tetrahedra in distorted MOF‐5 structures.

**Figure 24:**
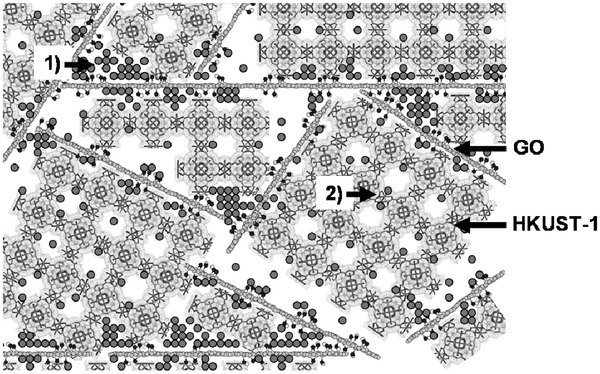
Visualization of the NH_3_ adsorption sites in the HKUST‐1 and GO composites: 1) physisorption at the interface between MOFs and layers of GO, and 2) binding to the copper centers of HKUST‐1 (NH_3_: dark gray circle). Reproduced with permission.^[^ ^119^ ^]^ Copyright 2010, American Chemical Society.

Similarly, composites of HKUST‐1 and GO (5%, 9%, 18%, 38%, and 46%) were studied by Bandosz and co‐workers for NH_3_ capture in both dry and humid conditions.^[^ ^119^, ^120^ ^]^ Unlike MOF‐5–GO, these composites were stable in humid conditions, as confirmed by PXRD. Moreover, the NH_3_ adsorption capacity of the composites surpassed the calculated adsorption capacity of the physically mixed components, indicating the synergetic effect in the composites (**Figure** **24**). One factor for the increased NH_3_ adsorption is the enhanced porosity and dispersive forces arising from the layers of graphene. The other factor is the unsaturated Cu sites in HKUST‐1. These mechanisms were additionally investigated based on the isosteric heats of adsorption calculated from isotherms, molecular simulations, and microcalorimetric analysis in the follow‐up studies.^[^ ^121^, ^122^ ^]^ Based on the heats of adsorption calculations, the adsorption affinity of NH_3_ to different adsorption sites in the composite was evaluated, and this result corresponded to the result obtained via molecular simulation. Additionally, the Lewis interaction energy between NH_3_ and the Cu sites was calculated to be 70–80 kJ mol^−1^, which is comparable to the interaction energy (70–100 kJ mol^−1^) of NH_3_ with the ligands of MOF.

Furthermore, composites of MIL‐100(Fe) and GO with different ratios of the two constituents were investigated by Petit and Bandosz.^[^ ^123^ ^]^ The fine structures of the composites were characterized by PXRD, N_2_ adsorption, differential thermal analysis, FT‐IR spectroscopy, Raman spectroscopy, and SEM. The results showed that fabricating well‐mixed composites of GO and MIL‐100(Fe) was unfavorable. The composite exhibited a reduced porosity and a lower NH_3_ adsorption capacity than those anticipated for the physically mixed MIL‐100(Fe) and GO. This could be explained based on the existence of layers of GO that interfere with the formation of spherical MOF frameworks after binding. Thus, studies related to MOF‐mixed‐composites for NH_3_ removal revealed that composite materials have strong advantages in terms of affording additional NH_3_ adsorption sites.

## 5 Conclusions and Outlook

**Figure 25:**
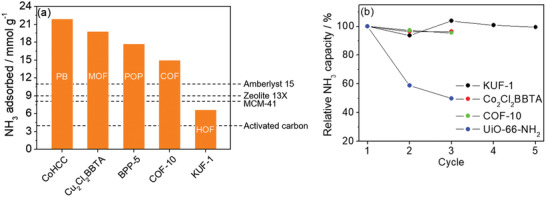
a) The highest NH_3_ capacity of the top‐performance adsorbent in each type of material at 298 K and 1 bar. The dashed lines indicate the capacity of conventional materials such as zeolite (Zeolite 13X), mesoporous silica (MCM‐41), activated carbon (Aldrich Darco 24226‐8), and polymer resin (Amberlyst 15). b) Recyclability of the high‐performance materials whose regeneration conditions were displayed in Table 1.

The removal or storage of NH_3_ has attracted substantial attention owing to the increasingly widespread usage of highly toxic gases. Among the diverse techniques for NH_3_ treatment, porous adsorbents are recognized as promising tools due to their versatile structures and adjustable pore characteristics. In this review, we introduced a variety of porous materials, including inorganic–organic hybrid materials such as MOFs, MOSs, and Prussian blues, and porous organic materials such as COFs, POPs, HOFs, activated carbons, and their composites with carbon, polymers, or GO. In practical applications, an ideal adsorbent should exhibit high chemical and thermal stability, superior NH_3_ uptake even in the presence of moisture, regeneration under mild conditions, and long‐term durability. As the polar and basic nature of NH_3_ could trigger the disintegration of frameworks, chemically stable platforms involving multivalent metal cations, multiply coordinated ligands, or strong covalent bonds are generally chosen for examination. For each material, the NH_3_ adsorption capacity was determined via NH_3_ isotherms or microbreakthrough measurements. The highest NH_3_ capacities of the top‐performance materials in each category are summarized in **Figure** **25**a. The adsorption capacity of most NH_3_ adsorbents surpasses that of conventional materials such as zeolite, mesoporous silica, activated carbon, and polymer resin. Some materials show recyclability although regeneration conditions are different from each material (Figure 25b).

Studies revealed that NH_3_ adsorption depends on the interplay among the density of active sites, the strength of adsorbent–adsorbate interactions, pore size, and effective surface area. To enhance the NH_3_ capture performance, Lewis acidic open metal sites were provided through the removal of residual solvents from the metal clusters of a MOF, resulting in a strong binding affinity with NH_3_ due to the acid–base interaction. Pore surface modulation with acidic functional groups (—OH, —NH_2_, —COOH, —SO_3_H, etc.) is another essential key to improving the adsorption capacity. Additionally, reducing the pore size could aid the capture of NH_3_ with small kinetic diameter of about 2.9 Å, whereas decorating the pore wall with overly bulky groups hindered access into the pore. As for adsorption under humid conditions, competitive adsorption between the H_2_O and NH_3_ molecules obstructed selective adsorption in some cases while the solubility of NH_3_ in H_2_O synergistically boosted adsorption in other cases. The stability of the structures could be supplemented by fabricating composites via postsynthetic coating or physical/chemical mixing.

Strong Lewis acidic sties, large surface areas, and different chemical environments in the pores are distinctive advantages of MOFs, while they display weak structural stabilities under NH_3_ conditions. Porous organic polymers composed of strong C—C bonds have positive traits of high structural stabilitiy and reusability for NH_3_ capture although their NH_3_ capacities are moderate. It is ideal that the prospective adsorbents should encompass both high NH_3_ capacity and structural integrity under exposure to realistic NH_3_ conditions. Thus, future researches on porous materials for NH_3_ capture provide adequate synthetic routes toward enhancing the performances in terms of the capacity and stability to meet the actual demand for NH_3_ removal applications.

As research on COFs, POPs, and HOFs is currently in the initial stage, extensive postmodification strategies exploiting their robust properties are anticipated. Moreover, more studies on shaping and processing the adsorbent powders for industrial applications are necessary. Regardless of their exceptional performances, the developed adsorbents will fail to secure their place in the market unless they are further engineered to minimize the production cost. Ultimately, advances in the development and optimization of prospective sorbents will eliminate the concerns accompanying their usage and promote the use of this valuable as with guaranteed safety to humanity and the environment.

## Conflict of Interest

The authors declare no conflict of interest.
